# Comparisons of infection events associated with tumor necrosis factor inhibitors in patients with inflammatory arthritis: A systematic review and network meta-analysis

**DOI:** 10.3389/fphar.2024.1376262

**Published:** 2024-07-11

**Authors:** Ziwei Jiang, Yue Zou, Guangyao Li, Sixuan Zhao, Wei Zhang

**Affiliations:** ^1^ Department of Pharmacy, Beijing Jishuitan Hospital, Capital Medical University, Beijing, China; ^2^ Department of Pharmacy, Beijing Tongren Hospital, Capital Medical University, Beijing, China

**Keywords:** tumor necrosis factor inhibitors, inflammatory arthritis, infection, network meta-analysis, systematic review

## Abstract

**Objective:** To compare the risk of infection in inflammatory arthritis patients treated with tumor necrosis factor (TNF) inhibitors.

**Methods:** PubMed, Embase, and the Cochrane Library were systematically searched from inception to 28 December 2023 for randomized controlled trials (RCTs) assessing TNF inhibitors and reporting infections. Subsequently, pairwise and network meta-analyses were conducted to determine odds ratios (ORs) and the corresponding 95% confidence intervals (CIs).

**Results:** A total of 61 RCTs involving 20,458 patients were included. Pairwise meta-analysis revealed that certolizumab pegol was significantly associated with an increased risk of serious infection compared to placebo (OR:2.28, 95% CI: 1.13–4.62). Both adalimumab and certolizumab pegol were also significantly associated with an increased risk of any infection compared to placebo (OR:1.18, 95% CI: 1.06 to 1.30 and OR:1.40, 95% CI: 1.11 to 1.76, respectively). Moreover, a network meta-analysis indicated that certolizumab pegol and infliximab were associated with a higher risk of serious infection compared to other TNF inhibitors. In the cumulative ranking of any infection risk, certolizumab pegol had the highest risk compared with others. TNF inhibitors increased the risk of tuberculosis but not that of herpes zoster.

**Conclusion:** Available evidence indicates etanercept and golimumab are likely associated with a lower risk of infection compared to other TNF inhibitors in inflammatory arthritis. For patients at a heightened risk of infection, prioritizing the use of etanercept and golimumab may be advisable to minimize patient risk.

**Systematic Review Registration:** identifier CRD42022316577

## 1 Introduction

Inflammatory arthritis (IA) is a heterogeneous pathology inducing motor system impairment, joint function loss, and joint ankylosis. Additionally, persistent inflammation may affect other organs, considerably impacting the quality of life. The global prevalence of inflammatory arthritis approximates 3% (Pfeffer et al., 1993; Fraenkel et al., 2021). Rheumatoid arthritis (RA), psoriatic arthritis (PsA), and ankylosing spondylitis (AS) are the most common subtypes of inflammatory arthritis.

Currently, multiple guidelines recommend prompt initiation of treatment with TNF inhibitors in individuals with rheumatoid arthritis, psoriatic arthritis, and ankylosing spondylitis who exhibit an inadequate response to standard or other conventional treatments (Singh et al., 2019; Ward et al., 2019; Smolen et al., 2020; Fraenkel et al., 2021). TNF inhibitors are effective therapeutics in inflammatory arthritis (Maini et al., 1999; Gorman et al., 2002; Weinblatt et al., 2003; Mease et al., 2005). Multiple studies have shown infection is the most common adverse event of TNF inhibitors (Burmester et al., 2013; Husni et al., 2022). This may result from the suppression of TNF-α-mediated immune responses, likely increasing the risk of infection. The developed infection may result in prolonged hospitalization or even be life-threatening.

Five TNF inhibitors have received FDA approval and are extensively used in the treatment of inflammatory arthritis, all of which target TNF-α, with etanercept additionally suppressing TNF-β. It remains controversial whether the five TNF inhibitors are different in terms of infection incidence. Previous reports have indicated TNF inhibitors do not differ in infection risk (Rubbert-Roth et al., 2018). However, the growing number of patients receiving TNF inhibitors has led to gradual observations of distinctions in infection risk among the five TNF inhibitors (Bongartz et al., 2006; Saliba et al., 2016).

Because of the absence of head-to-head studies, direct evidence is lacking to compare the current TNF inhibitors for both efficacy and safety. Notably, the first large-scale study was published directly comparing adalimumab and certolizumab pegol for efficacy and safety, with comparable incidence rates of infection, tuberculosis, and opportunistic infections among them in 2016 (Smolen et al., 2016). However, it is crucial to acknowledge that this is the sole study providing a direct comparison, and large-scale data comparisons are currently insufficient. Consequently, we aimed to assess the risk of infection associated with TNF inhibitors by network meta-analysis to select drugs with a lower risk of infection and provide medication suggestions for susceptible patients.

## 2 Materials and methods

This network meta-analysis was conducted based on the Preferred Reporting Items for Systematic Reviews and Meta-analyses (PRISMA) statement complemented with the PRISMA extension for NMAs (Hutton et al., 2015; Page et al., 2021) and has been registered with PROSPERO (No. CRD42022316577).

### 2.1 Search strategy and study selection

PubMed, Embase and the Cochrane Library were systematically searched to collect relevant studies assessing TNF inhibitors for the treatment of inflammatory arthritis from inception to 27 December 2023. The literature search was restricted to human studies published in English. Our English search terms encompassed “Adalimumab,” “Etanercept,” “Infliximab,” “Golimumab,” “Certolizumab pegol,” “Rheumatoid arthritis,” “Ankylosing spondylitis,” “Psoriatic arthritis” and “Randomized Controlled Trial.” The specific search strategy is detailed in Supplementary Table S1. Additionally, we identified further potential trials by manual searching the references of the included trials and relevant meta-analyses. Two reviewers (ZJ and YZ) selected the included studies according to the following criteria: 1) patients received the recommended standard dosages of TNF inhibitors (adalimumab, etanercept, golimumab, infliximab, and certolizumab) (see Table 1 for specific details). In patients administered background therapy, the latter was required to be the same; 2) primary outcomes included serious infection (defined as a diagnosis of infection requiring antimicrobial therapy and/or hospitalization) and any infection; secondary outcome measures included opportunistic infection (including *Candida* spp., *Pseudomonas* spp., *Pneumocystis* spp., *etc.*, excluding tuberculosis infection), tuberculosis (including pulmonary tuberculosis, peritoneal tuberculosis, disseminated tuberculosis, *etc.*) and herpes zoster; 3) study design was RCT.

**TABLE 1:** Characteristics of the RCTs included in the meta-analysis.

Author	Study ID ClinicalTrial. Gov	Disease	Intervention	Dosage	Number of patients	Follow-up	Age (years)	Duration of Disease (years)
Weinblatt ME 1999	NR	RA	etanercept	25 mg twice weekly	89	24 weeks	50	13
Lipsky PE 2000	NR	RA	infliximab	3 mg/kg at 0.2, and 6 weeks, then every 8 weeks	174	54 weeks	52.5	10.5
Braun J 2002	NR	AS	infliximab	5 mg/kg at 0.2, and 6 weeks	69	12 weeks	39.8	15.6
Gorman JD 2002	NR	AS	etanercept	25 mg twice weekly	40	4 months	38.5	13.5
Davis JC 2003	NR	AS	etanercept	25 mg twice weekly	277	24 weeks	42.0	10.4
Furst DE 2003	NR	RA	adalimumab	40 mg every other week	636	24 weeks	55.4	10.4
Keystone EC 2004	NR	RA	adalimumab	40 mg every other week	407	52 weeks	50.5	10.9
St Clair EW 2004	NR	RA	infliximab	3 mg/kg at 0.2, and 6 weeks, then every 8 weeks	641	54 weeks	56.1	0.8
Mease PJ 2004	NR	PsA	etanercept	25 mg twice weekly	205	24 weeks	47.4	9.1
Marzo-Ortega H 2005	NR	AS	infliximab	5 mg/kg at 0.2, and 6 weeks, then every 8 weeks	42	30 weeks	Infliximab:41* Placebo:39*	Infliximab:8* Placebo:10*
Mease PJ 2005	NR	PsA	adalimumab	40 mg every other week	313	24 weeks	49	9.5
van der Heijde D 2005	NR	AS	infliximab	5 mg/kg at 0.2, and 6 weeks, then every 6 weeks	279	24 weeks	Infliximab:40* Placebo:41*	Infliximab:7.7* Placebo:13.2*
van der Heijde D 2006a	NR	AS	etanercept	25 mg twice weekly and 50 mg once weekly	356	12 weeks	40.6	9.4
van der Heijde D 2006b	NR	AS	adalimumab	40 mg every other week	315	24 weeks	42.3	10.9
Abe T 2006	NR	RA	infliximab	3 mg/kg at 0.2, and 6 weeks, then every 8 weeks	96	14 weeks	55.2	8.3
Westhovens R 2006	NR	RA	infliximab	3 mg/kg at 0.2, and 6 weeks, then every 8 weeks	723	22 weeks	Infliximab:53* Placebo:52*	Infliximab:7.8* Placebo:8.4*
Zhang FC 2006	NR	RA	infliximab	3 mg/kg at 0.2, and 6 weeks, then every 8 weeks	105	18 weeks	48.4	7.5
van der Heijde D 2007	NCT00393471	RA	etanercept	25 mg twice weekly	459	3 years	52.8	6.8
Genovess MC 2007	NR	PsA	adalimumab	40 mg every other week	100	12 weeks	49.1	7.4
Kay J 2008	NR	RA	golimumab	50 mg every 4 weeks	71	20 weeks	Golimumab:57* Placebo:52*	Golimumab:8.2* Placebo:5.6*
Emery P 2008	NR	RA	etanercept	50 mg once weekly	528	52 weeks	51.4	9.0
Keystone E 2008	NCT00152386	RA	certolizumab pegol	400 mg initially and at weeks 2, and 4, followed by 200 mg every other week	591	52 weeks	52.3	6.2
Inman RD 2008	NCT00265083	AS	golimumab	50 mg every 4 weeks	215	14 weeks	39.7	5.9
Miyasaka N 2008	NR	RA	adalimumab	40 mg every other week	178	24 weeks	55.2	9.2
Schiff M 2008	NCT00095147	RA	infliximab	3 mg/kg at 0, 2, 6, and 17 weeks, then every 8 weeks	275	28 weeks	49.2	7.7
Bejarano V 2008	NR	RA	adalimumab	40 mg every other week	148	56 weeks	47	0.7
Chen DY 2009	NR	RA	adalimumab	40 mg every other week	47	12 weeks	53	6.7
Kavanaugh A 2009	NCT00265096	PsA	golimumab	50 mg every 4 weeks	160	24 weeks	46.3	7.4
Emery P 2009	NR	RA	golimumab	50 mg every 4 weeks	318	24 weeks	49.7	3.2
Fleischmann R 2009	NCT00548834	RA	certolizumab pegol	400 mg every 4 weeks	220	24 weeks	53.8	9.5
Combe B 2009	NR	RA	etanercept	25 mg twice weekly	151	2 years	51	NR
Keystone EC 2009	NR	RA	golimumab	50 mg every 4 weeks	222	24 weeks	52	5.7
Smolen J 2009	NCT00175877	RA	certolizumab pegol	400 mg initially and at weeks 2, and 4, followed by 200 mg every other week	373	24 weeks	51.8	5.9
Smolen JS 2009	NCT00299546	RA	golimumab	50 mg every 4 weeks	307	24 weeks	54.3	9.7
van Vollenhoven RF 2011	NCT00595413	RA	adalimumab	40 mg every other week	155	25 weeks	53.5	8.6
Choy E 2012	NCT00544154	RA	certolizumab pegol	400 mg every 4 weeks	247	24 weeks	54.3	9.6
Tanaka Y 2012	NCT00727987	RA	golimumab	50 mg every 4 weeks	174	24 weeks	50.8	8.7
Kavanaugh A 2013	NCT00420927	RA	adalimumab	40 mg every other week	1,032	26 weeks	50.5	4.3
Fleischmann R 2012	NCT00550446	RA	adalimumab	40 mg every other week	112	24 weeks	53.5	9.3
van Vollenhoven RF 2012	NCT00853385	RA	adalimumab	40 mg every other week	312	3 months	52.9	8
Baranauskaite AT 2012	NCT00367237	PsA	infliximab	5 mg/kg at 0.2, and 6 weeks, then every 6 weeks	111	16 weeks	41.2	3.2
Weinblatt ME 2012	NCT00717236	RA	certolizumab pegol	400 mg initially and at weeks 2, and 4, followed by 200 mg every other week	1,063	12 weeks	55	8.7
Smolen JS 2013	NCT00565409	RA	etanercept	50 mg once weekly	402	52 weeks	48.2	7
Detert J 2013	NR	RA	adalimumab	40 mg every other week	172	48 weeks	49.8	0.1
Takeuchi T 2013	NR	RA	golimumab	50 mg every 4 weeks	206	16 weeks	52.6	9.3
Takeuchi T 2014	NCT00870467	RA	adalimumab	40 mg every other week	334	26 weeks	54	0.3
Yamamoto K 2014	NCT00791921	RA	certolizumab pegol	400 mg initially and at weeks 2, and 4, followed by 200 mg every other week	230	24 weeks	55.7	5.6
Huang F 2014	NCT01114880	AS	adalimumab	40 mg every other week	344	12 weeks	29.9	3
Smolen JS 2015	NCT00674362	RA	certolizumab pegol	400 mg initially and at weeks 2, and 4, followed by 200 mg every other week	194	52 weeks	53.8	4.6
Hobbs K 2015	NCT01313208	RA	etanercept	50 mg once weekly	210	12 weeks	56	7.9
Li Z 2016	NCT01248780	RA	golimumab	50 mg every 4 weeks	263	24 weeks	47.2	7.8
Smolen JS 2016	NCT01500278	RA	Adalimumab, certolizumab pegol	40 mg every other week, 400 mg initially and at weeks 2, and 4, followed by 200 mg every other week	1,039	104 weeks	53.2	5.9
Taylor PC 2017	NCT01710358	RA	adalimumab	40 mg every other week	818	52 weeks	53	10
Kang YM 2018	NCT00993317	RA	certolizumab pegol	400 mg given at 0,2,4, with a subsequent dosage of 200 mg given every 2 weeks	127	24 weeks	51.3	6.2
van der Heijde 2018	NCT02696785	AS	adalimumab	40 mg every other week	176	16 weeks	42.2	7.2
Bi L 2019	NCT02151851	RA	certolizumab pegol	400 mg initially and at weeks 2, and 4, followed by 200 mg every other week	429	24 weeks	47.9	6.9
Fleischmann R 2019	NCT02629159	RA	adalimumab	40 mg every other week	979	26 weeks	54	8
Cohen S 2020	NCT02833350	RA	adalimumab	40 mg every other week	221	12 weeks	50	5
Mclnnes IB 2021	NCT03104400	PsA	adalimumab	40 mg every other week	852	24 weeks	50.9	6.0
Smolen JS 2022	NCT02760407	RA	adalimumab	40 mg every other week	705	24 weeks	54.3	7.2
McInnes IB 2023	NCT03895203	PsA	adalimumab	40 mg every other week	421	16 weeks	48.8	5.8

^a^ Asterisks indicate the median.

^b^ Abbreviation: NR, not reported; RA, rheumatoid arthritis; AS, ankylosing spondylitis; PsA, psoriatic arthritis.

### 2.2 Data extraction and quality assessment

Two reviewers (ZJ and YZ) independently performed the literature search and data extraction. In case of disagreement, a third reviewer (GL) was involved in arbitration. Missing data were diligently sought as much as possible by contacting the corresponding author.

After screening the literature, the titles and abstracts were first read to exclude irrelevant studies. Then, full texts were further read for final inclusion. The data extracted mainly included: 1) basic information about the included studies (study title, publication year, country, and NCT number); 2) basic patient characteristics (age and gender); 3) sample size; 4) key information for risk of bias assessment; 5) information related to the outcome measures.

Two investigators (ZJ and YZ) independently assessed the risk of bias of all included studies with the Cochrane risk of bias tool (Cumpston et al., 2019). In case of disagreement, a third investigator (GL) was involved in arbitration. The quality evaluation included random sequence generation (selection bias), allocation concealment (selection bias), blinding (performance and detection biases), incomplete data (attrition bias), selective reporting (reporting bias), and other biases. We used the Grading of Recommendations, Assessment, Development, and Evaluation (GRADE) approach to assess the certainty of the evidence (Puhan et al., 2014).

### 2.3 Statistical analysis

In pairwise meta-analysis, the random-effects model was used to determine odds ratios (ORs) and 95% confidence intervals (CIs) for the risk of infection. The degree of heterogeneity was assessed by the I^2^ value, with I^2^ < 25% considered as no heterogeneity, 25% ≤ I^2^ < 50% as low heterogeneity, 50% ≤ I^2^ < 75% as moderate heterogeneity, and I^2^ ≥ 75% as high heterogeneity (Higgins et al., 2003). The risk of infection for the five TNF inhibitors was preliminarily predicted by pairwise comparison. To assess the robustness of the primary results, sensitivity analysis was performed by excluding trials with a follow-up ⩾52 weeks and a sample size <50. Funnel plots and Egger’s test were used to assess publication bias (Sterne et al., 2011).

To further compare the risk of infection for the five TNF inhibitors, a network meta-analysis was performed with the random-effects model using the “mvmeta” command. ORs and 95% CIs were obtained for different interventions. The ranking of TNF inhibitor infection risk was assessed by surface under the cumulative ranking curve (SUCRA) analysis (Shim et al., 2017). Inconsistency tests were performed to check for discrepancies between direct and indirect evidence in closed loops (Song et al., 2011). Small sample effects were assessed by comparing adjusted funnel plots (Chaimani and Salanti, 2012). Additionally, meta-regression and subgroup analyses were utilized to examine the effects of covariates, including trial features (disease type, follow-up time, age, and background therapy) on the pooled effect size. Statistical analysis was carried out with RevMan 5.4 (The Cochrane Collaboration, 2020) and STATA 17 (Stata Corp., College Station, Texas).

## 3 Results

### 3.1 Study selection and study characteristics

**FIGURE 1:**
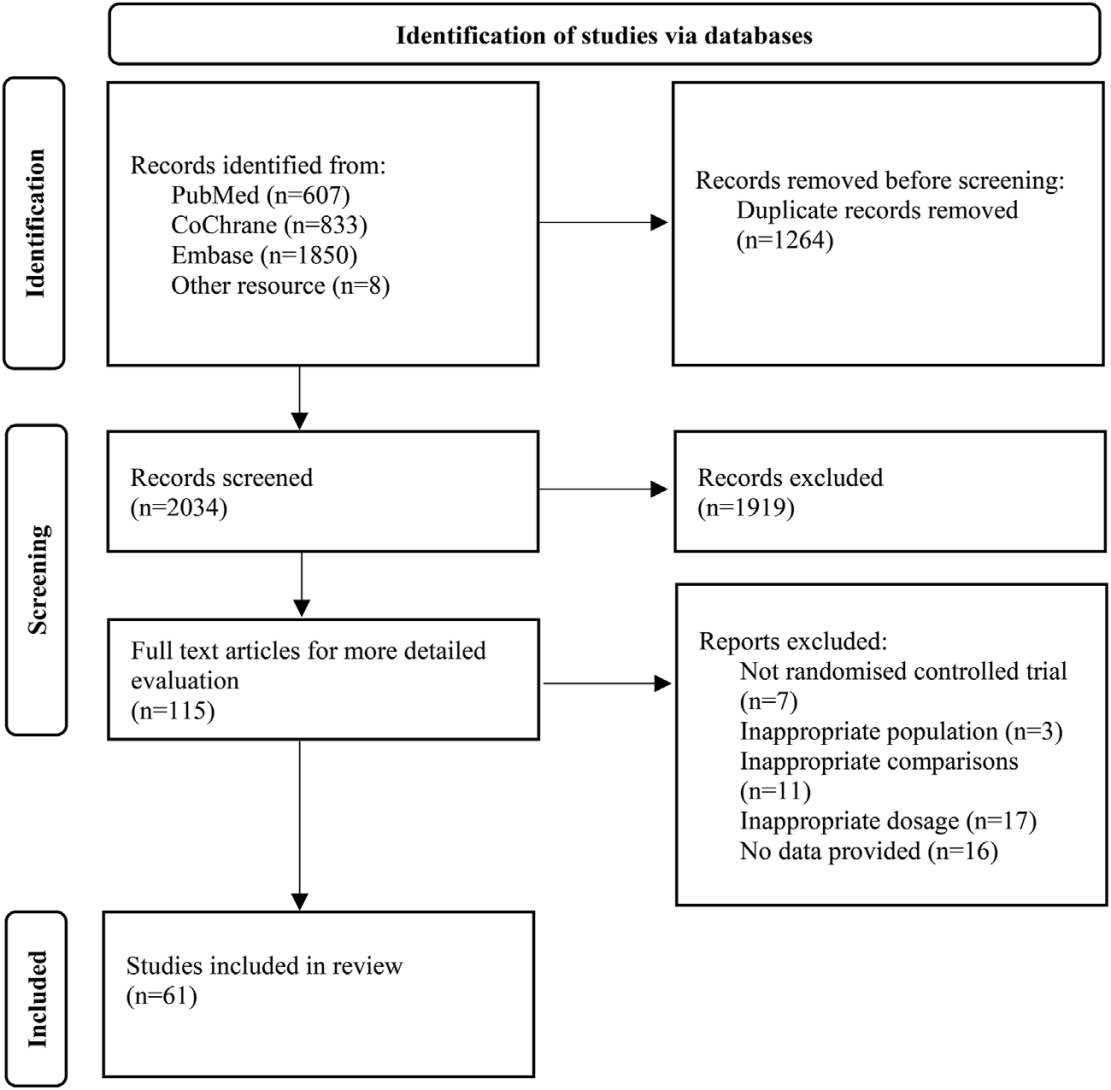
Flow diagram based on PRISMA guidelines.

**FIGURE 2:**
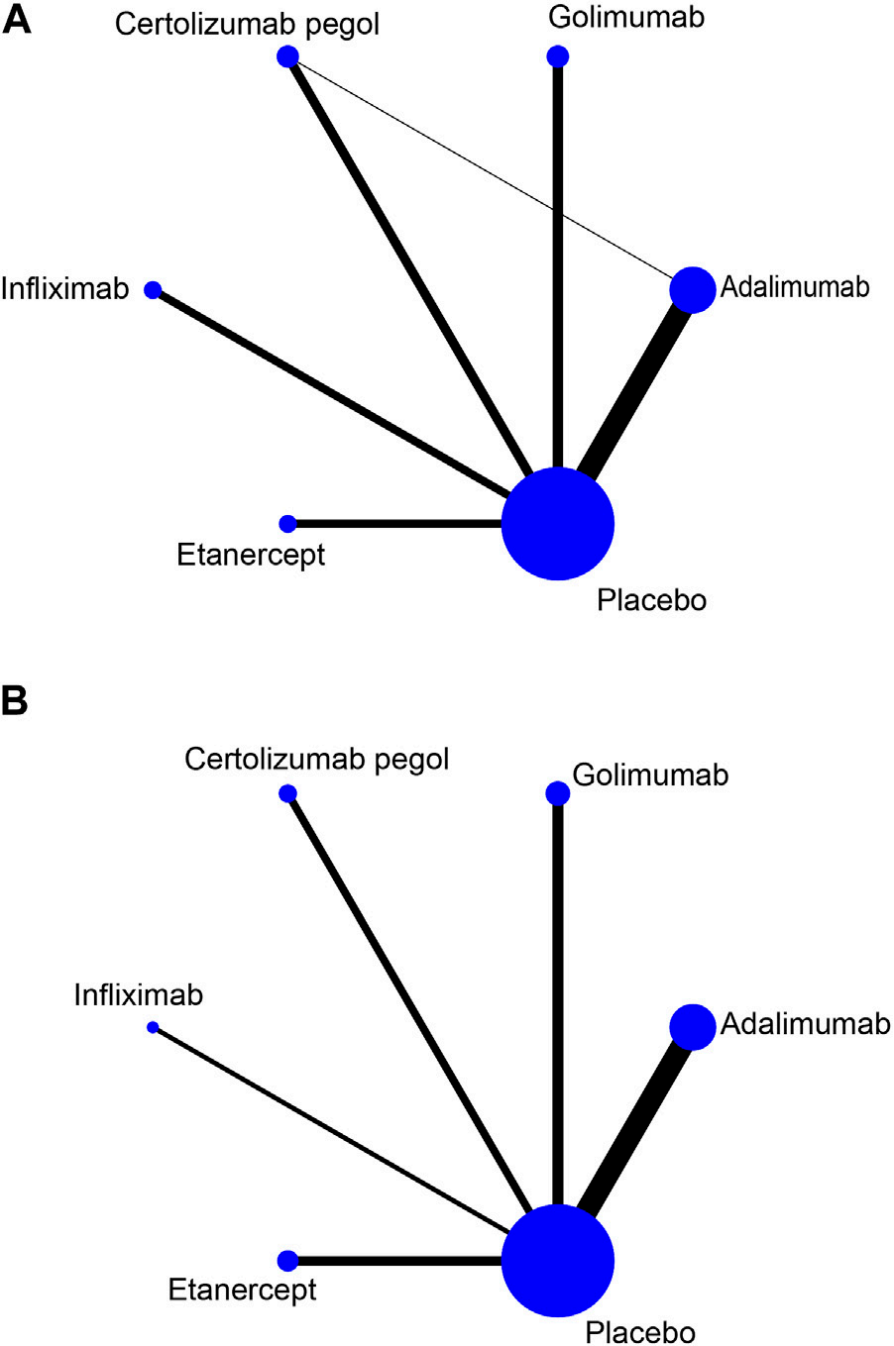
Network plots for TNF inhibitors and the risk of infection. The node size reflects the number of patients in the treatment group and the line thickness reflects the number of trials for the given comparison. No connecting line between the two treatments indicates no direct comparison **(A)** Risk of serious infection. **(B)** Risk of any infection.

Upon systematic search, 3,298 records were retrieved; of these, 1,264 repeated records were eliminated and 61 were finally included based on title, abstract, and full-text reading. The study flowchart is depicted in Figure 1. Available direct and indirect comparisons are represented by network plots (Figure 2). This study included 20,458 patients, 16,183 with rheumatoid arthritis, 2,113 with ankylosing spondylitis and 2,162 with psoriatic arthritis. Basic characteristics are shown in Table 1. Totally 61 studies were included by random grouping. Among them, three included trials did not perform blinding, resulting in a judgment of high risk of bias. A trial utilized single blinding. None of the included studies reported concealment, incomplete data, or selective reporting of outcome measures with other risks of bias. The literature quality is shown in Supplementary Figure S1.

### 3.2 Pairwise meta-analysis

Pairwise meta-analysis indicated that certolizumab pegol was significantly associated with an increased risk of serious infection compared to placebo (OR:2.28, 95%CI:1.13–4.62). Conversely, no significant differences were detected between adalimumab (OR:1.27, 95%CI:0.85–1.88), etanercept (OR:0.79, 95%CI:0.46–1.34), golimumab (OR:0.69, 95%CI:0.36–1.35), infliximab (OR:1.39, 95%CI:0.72–2.68) and placebo. Notably, both adalimumab and certolizumab pegol were associated with a significantly higher risk of any infection compared to placebo (OR:1.18, 95%CI:1.06 to 1.30 and OR:1.40, 95%CI:1.11 to 1.76, respectively).

Our analysis revealed no significant association between TNF inhibitors and opportunistic infections. However, in terms of tuberculosis, TNF inhibitors significantly increased the risk of infection compared to placebo (OR:2.21, 95%CI:1.05–4.66). Conversely, in terms of herpes zoster, TNF inhibitors were not significantly associated with the risk of infection compared to placebo (OR:1.50, 95%CI:0.72–3.11).

Overall, no significant heterogeneity was observed, as indicated in Supplementary Figure S2. Furthermore, no significant publication bias was detected by Egger’s test (*p* > 0.05), and visual inspection of funnel plots was carried out for the four outcome measures (see Supplementary Figure S3). Sensitivity analysis, detailed in Supplementary Figure S4, did not significantly alter the main results, suggesting robustness in our findings.

### 3.3 Network meta-analysis

**FIGURE 3:**
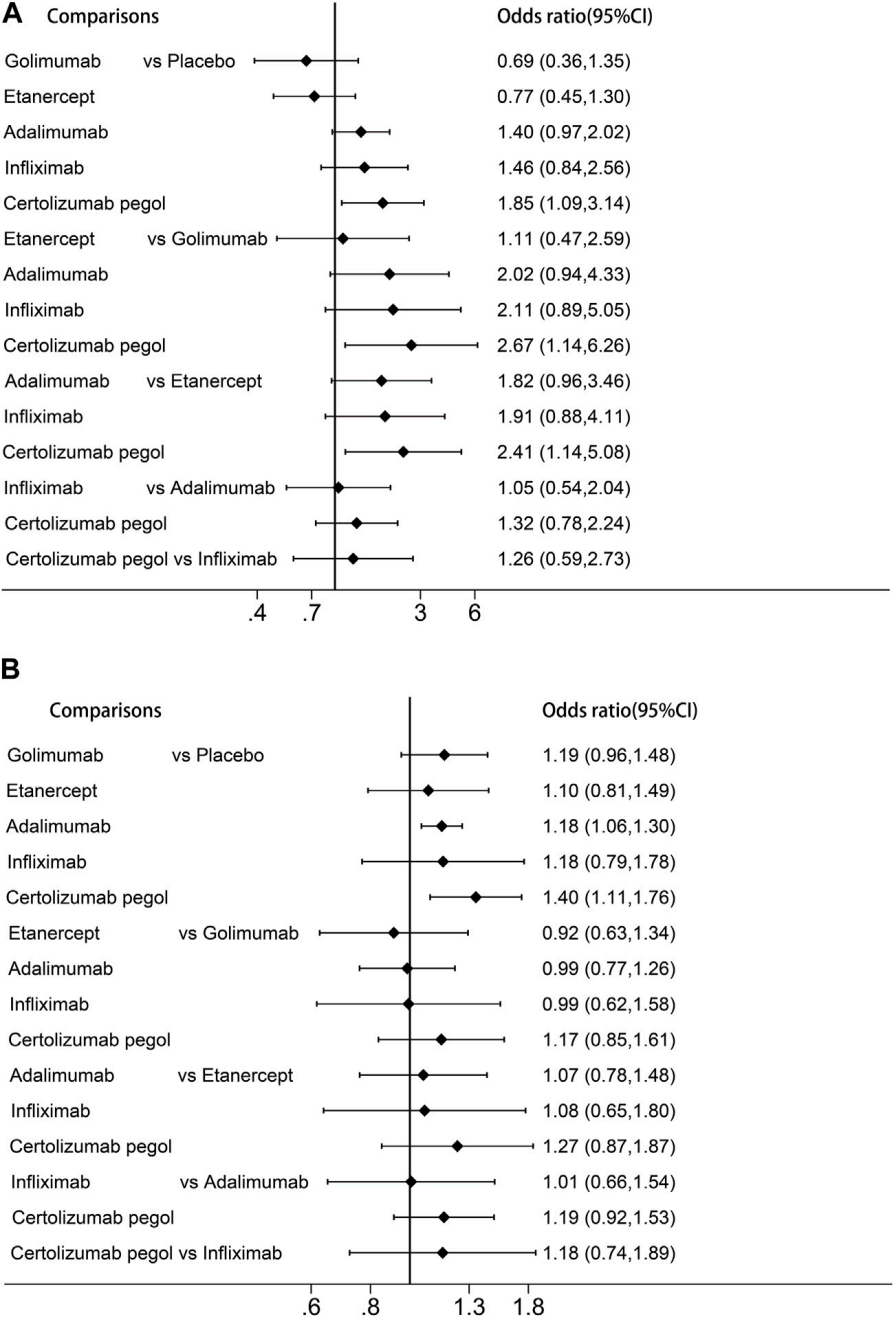
Forest plots of the network meta-analysis **(A)** Risk of serious infection. **(B)** Risk of any infection.

In network meta-analysis, certolizumab pegol had a significantly increased risk of serious infection in comparison with placebo, golimumab, and etanercept (OR:1.85, 95%CI:1.09 to 3.14; OR:2.67, 95%CI:1.14 to 6.26 and OR:2.41, 95%CI:1.14 to 5.08, respectively; Figure 3). We performed a ranking of infection risk based on the surface under the cumulative ranking curves (Supplementary Figure S5). The cumulative probability ranking for the risk of serious infection was highest for certolizumab pegol (90.7%), followed by infliximab (73.1%), adalimumab (69.5%), etanercept (17.0%) and golimumab (12.8%). Direct and indirect comparisons revealed no significant differences (*p* = 0.377).

For any infection, adalimumab was associated with a significantly higher risk compared to placebo (OR:1.18, 95%CI:1.06–1.30). The results of the cumulative ranking of any infection risk demonstrated certolizumab pegol had the highest risk of infection (87.7%), followed by golimumab (56.5%), infliximab (53.3%), adalimumab (53.6%) and etanercept (38.1%). Adjusted funnel plots for comparison indicated the absence of publication bias and a low sample effect, as illustrated in Supplementary Figure S3. However, due to limited data availability, network meta-analyses could not be performed for opportunistic infections, herpes zoster infection, and tuberculosis.

### 3.4 Meta-regression and subgroup analysis

A meta-regression analysis was performed based on disease type, follow-up time, age, and background therapy. The results showed that the latter factors may had no significant effects on the results (*p* > 0.05) (Supplementary Table S2). Meanwhile, detailed subgroup analyses were carried out and no possible influencing factors were found (Supplementary Table S3).

The GRADE assessments are presented in the Supplementary Table S4. As all included studies were RCTs, the quality of starting evidence was high. We performed sensitivity analyses that did not identify any studies with a high risk of bias, indicating that there is no need to downgrade because of risk of bias. In addition, most comparisons were downgraded due to imprecisions. Consequently, the quality of our comparisons was moderate.

## 4 Discussion

In this study, we evaluated of the infection risk among patients with inflammatory arthritis receiving treatment with five different TNF inhibitors. The results demonstrated all TNF inhibitors increased the risk of any infection, with certolizumab pegol displaying the highest risk. Etanercept and golimumab had a lower risk of serious infection. TNF inhibitors increased the risk of tuberculosis but not that of opportunistic infections and herpes zoster. In addition, we performed a detailed meta-regression analysis to assess the effects of the five TNF inhibitors on the risk of infection for various disease types, ages, and background therapies. None of these factors affected the results.

Several studies have shown that TNF inhibitors significantly increase the risk of serious infections and any infection (Minozzi et al., 2016; Chiu and Chen, 2020). Our conclusions are generally aligned with previous studies. In terms of serious infections, patients using adalimumab, certolizumab, and infliximab had an increased risk of serious infections in a study comparing safety in rheumatoid arthritis. A similar trend was observed for golimumab in combination with methotrexate. In contrast, serious infection rates tended to be lower with etanercept (Michaud et al., 2014). Data from the Dutch Rheumatoid Arthritis Monitoring (DREAM) registry revealed that patients with rheumatoid arthritis treated with etanercept had a lower risk of serious infection than adalimumab and infliximab (van Dartel et al., 2013). In a head-to-head clinical trial directly comparing the efficacy and safety of certolizumab and adalimumab in patients with rheumatoid arthritis, it was found that there was no significant difference in the occurrence of serious infections between adalimumab and certolizumab (Smolen et al., 2016). Galloway et al. (2011) concluded that the highest risk of infection occurs within the first 6 months of treatment, stabilizing between 24 and 36 months. Factors such as a history of serious infections, glucocorticoid dose, smoking, diabetes, and older age were identified as significant predictors of serious infections in patients treated with biologics (Singh, 2016). Therefore, extra caution should be exercised regarding the occurrence of serious infections in these patients. In terms of any infection, a meta-analysis included 71 RCTs involving 22,760 adult rheumatology patients and seven open-label extension (OLE) studies involving 2,236 patients found that TNF inhibitors significantly increased the risk of any infection (Minozzi et al., 2016). Overall, these findings and our study suggest that etanercept is a potentially safer option for infections, followed by golimumab.

Patients administered TNF inhibitors for immune-mediated inflammatory disorders are known to be susceptible to infection by diverse opportunistic pathogens, including *Coccidioides, Histoplasma, Nontuberculous mycobacteria*, and *Mycobacterium tuberculosis* (Winthrop and Chiller, 2009). Many trials included in published meta-analyses have demonstrated that TNF-α inhibitors significantly increase the risk of tuberculosis in patients with rheumatic diseases (RA, AS, PsA) (Ai et al., 2015; Minozzi et al., 2016). Related reports have further identified structural and functional differences between antibodies and soluble receptors, resulting in distinct affinities for binding to TNF-α and effects on T cell proliferation and apoptosis. Additionally, etanercept slightly affects membrane-bound TNF, with a lower risk of tuberculosis compared with monoclonal antibodies (Dixon et al., 2010; Marotte and Cimaz, 2014). As a result, physicians must screen for primary tuberculosis or latent tuberculosis infection (LTBI) before initiating TNF-α therapy to decrease the risk of LTBI reactivation (Gardam et al., 2003; Fonseca et al., 2008). In patients with LTBI or previously treated tuberculosis, etanercept rather than other TNF inhibitors is recommended to reduce the risk of tuberculosis infection (Godfrey and Friedman, 2019).

Herpes zoster results from varicella-zoster virus (VZV) reactivation (Schmader and Dworkin, 2008). Currently, whether TNF inhibitors increase the risk of herpes zoster remains controversial. Contrary to some retrospective studies that suggested an association, our study did not find that the use of TNF inhibitors significantly increased the risk of herpes zoster infection. A retrospective study on the treatment of psoriatic arthritis and the risk of herpes zoster included 3,128 patients to explore the association of traditional antirheumatic drugs and TNF inhibitors with herpes zoster (Schmader and Dworkin, 2008). Conversely, several meta-analyses have also confirmed that TNF inhibitors increase the risk of herpes zoster infection, especially monoclonal antibodies (Strangfeld et al., 2009; Che et al., 2014; Redeker et al., 2022). We consider that the discrepancy may be related to the duration of follow-up. Studies have shown that the average time between initiation of TNF inhibitor therapy and the occurrence of a herpes zoster event of 8–19 months, with incidence peaking in the first 2 years after initiating biologic therapy (Zisman et al., 2016). In contrast, the trials we included had study durations of mostly 6 months, which prevented some events outside the study duration from being recorded. In 2021, the ACIP in the United States updated vaccine recommendations to administer two doses of recombinant herpes zoster vaccine (RZV) to prevent herpes zoster and its complications in adults ≥19 years who are or may be immunodeficient or immunosuppressed due to disease or therapy (Anderson et al., 2022).

We consider the difference in infection risk for TNF inhibitors to be mostly explained by their different molecular structures, leading to differences in pharmacokinetics and mechanisms of action (Wallis, 2009). Firstly, the difference in affinity among various TNF inhibitors is obvious. Certolizumab pegol improves pharmacokinetics, increases affinity, and prolongs half-life by forming pegylated structures (Pasut, 2014). It binds TNF with a higher affinity than other TNF inhibitors. The stronger affinity may predict more effective TNF inhibition. Secondly, the direct cytotoxicity of certolizumab differs from that of other TNF inhibitors in that it directly induces the death of transmembrane cells expressing TNF-α (Ueda et al., 2013). Finally, monoclonal antibodies represented by infliximab can also directly eliminate activated T cells and monocytes/macrophages by cell lysis or induction of apoptosis (Furst et al., 2006). These aspects could explain the trend that certolizumab and infliximab seem to be more susceptible to infection.

The strength of this study is the use of a network meta-analysis to assess risk differences between TNF inhibitors in the absence of direct head-to-head studies. Moreover, this network meta-analysis provided sufficient evidence for a relationship between TNF inhibitors and infection. However, this study had some limitations. First, we included RA, AS, PsA patients, which allowed the inclusion of more trials but may also increase the risk of bias. However, we conducted a meta-regression analysis to demonstrate the current conclusions may be applied to all patients with rheumatic disorders. Second, most evidence came from randomized controlled trials versus placebo, with only one head-to-head study. To generate more accurate results, more head-to-head trials are required. Third, all the studies had a certain time frame, so some infections that occurred after the study may not have been captured. Fourth, although the definitions in different versions of MedDRA may vary, this does not affect the accuracy of our results. Finally, rheumatoid arthritis had the most studies, while ankylosing spondylitis and psoriatic arthritis had relatively fewer studies. Therefore, rheumatoid arthritis may have a more significant impact on the final safety results.

## 5 Conclusion

In summary, this network meta-analysis showed that both golimumab and etanercept might have a lower risk of infection compared with other TNF inhibitors. These findings offer a foundation for drug selection in susceptible patients, aiming to reduce the risk of infection and promote individualized medication.

## Data Availability

The original contributions presented in the study are included in the article/Supplementary Material, further inquiries can be directed to the corresponding author.
