# Supplementary material for: Comparisons of infection events associated with tumor necrosis factor inhibitors in patients with inflammatory arthritis: A systematic review and network meta-analysis
Source: Front Pharmacol. 2024 Jul 11;15:1376262. doi: 10.3389/fphar.2024.1376262 (PMC11273365; doi:10.3389/fphar.2024.1376262)
Supplement: Supplementary file 1 [file DataSheet1.docx]

Supplementary Material

**Supplementary Table S1. Search Strategy**

**Supplementary Appendix S1. References for included trials.**

**Supplementary Figure S1. Risk of bias assessments for each study based on adjusted Cochrane risk of bias tool.**

**Supplementary Figure S2. Pairwise meta-analysis of the effect of tumor necrosis factor-α inhibitors on the risk of infection.**

**Supplementary Figure S3. The publication bias assessment using comparison-adjusted funnel-plot and funnel-plot.**

**Supplementary Figure S4. Sensitivity analysis by excluding trials with follow-up ⩾52weeks and sample size <50.**

**Supplementary Figure S5. The SUCRA probabilities of the tumor necrosis factor-α inhibitors on risk of infections.**

**Supplementary Table S2.** **Meta-regression of trails’ characteristics on the risk of TNF inhibitors.**

**Supplementary Table S3.** **Subgroup analysis of trails’ characteristics.**

**Supplementary Table S4. GRADE assessment**

**Supplementary Table S5. Checklist of the PRISMA extension for network meta-analysis.**

**Supplementary Table S1. Search Strategy**

**Search date: December 28, 2023**

| **Data source** | **Search terms** |
| --- | --- |
| **PubMed** | ((((Controlled Clinical Trial) OR (Randomized Controlled Trial) OR (Controlled Clinical Trial) OR (Randomized Controlled Trial) OR RCT OR random* OR trial)  AND (infect*))  AND ((((((((((((((((TNF-α inhibitor[Title/Abstract]) OR (Tumor necrosis factor-α (TNF-α) inhibitor[Title/Abstract])) OR (anti-tumor necrosis factor-α (TNFα[Title/Abstract]))) OR (antitumor necrosis factor (TNF[Title/Abstract]))) OR (anti-TNF-α[Title/Abstract])) OR (TNF blocker[Title/Abstract])) OR (TNFalpha inhibitor[Title/Abstract])) OR (TNF-alpha inhibitor[Title/Abstract])) OR (Tumor Necrosis Factor alpha inhibitor[Title/Abstract])) OR (Anti-TNFalpha[Title/Abstract])) OR (Anti-tumor necrosis factor-alpha[Title/Abstract])) OR ((((("Adalimumab"[MeSH]) OR (Adalimumab[Title/Abstract])) OR (Humira[Title/Abstract])) OR (D2E7 Antibody[Title/Abstract])) OR (Antibody, D2E7[Title/Abstract]))) OR ((((((((((((("Etanercept"[MeSH]) OR (Etanercept[Title/Abstract])) OR (TNFR-Fc Fusion Protein[Title/Abstract])) OR (Fusion Protein, TNFR-Fc[Title/Abstract])) OR (TNFR Fc Fusion Protein[Title/Abstract])) OR (TNR 001[Title/Abstract])) OR (TNT Receptor Fusion Protein[Title/Abstract])) OR (TNR-001[Title/Abstract])) OR (TNF Receptor Type II-IgG Fusion Protein[Title/Abstract])) OR (TNF Receptor Type II IgG Fusion Protein[Title/Abstract])) OR (Recombinant Human Dimeric TNF Receptor Type II-IgG Fusion Protein[Title/Abstract])) OR (Recombinant Human Dimeric TNF Receptor Type II IgG Fusion Protein[Title/Abstract])) OR (Enbrel[Title/Abstract]))) OR ((((("Certolizumab pegol"[MeSH]) OR (Certolizumab pegol[Title/Abstract])) OR (Cimzia[Title/Abstract])) OR (CDP870[Title/Abstract])) OR (CDP 870[Title/Abstract]))) OR ((((("golimumab" [Supplementary Concept]) OR (Golimumab[Title/Abstract])) OR (CNTO-148[Title/Abstract])) OR (CNTO 148[Title/Abstract])) OR (Simponi[Title/Abstract]))) OR (((((("Infliximab"[MeSH]) OR (Infliximab[Title/Abstract])) OR (Monoclonal Antibody cA2[Title/Abstract])) OR (cA2, Monoclonal Antibody[Title/Abstract])) OR (MAb cA2[Title/Abstract])) OR (Remicade[Title/Abstract]))))  AND (((Rheumatoid Arthritis[Title/Abstract]) OR ((((((((((((((((((((("Spondylitis, Ankylosing"[Mesh]) OR (Spondylitis, Ankylosing[Title/Abstract])) OR (Spondyloarthritis Ankylopoietica[Title/Abstract])) OR (Ankylosing Spondylarthritis[Title/Abstract])) OR (Ankylosing Spondylarthritides[Title/Abstract])) OR (Spondylarthritides, Ankylosing[Title/Abstract])) OR (Spondylarthritis, Ankylosing[Title/Abstract])) OR (Ankylosing Spondylitis[Title/Abstract])) OR (Spondylarthritis Ankylopoietica[Title/Abstract])) OR (Bechterew Disease[Title/Abstract])) OR (Bechterew's Disease[Title/Abstract])) OR (Bechterews Disease[Title/Abstract])) OR (Marie-Struempell Disease[Title/Abstract])) OR (Marie Struempell Disease[Title/Abstract])) OR (Rheumatoid Spondylitis[Title/Abstract])) OR (Spondylitis, Rheumatoid[Title/Abstract])) OR (Spondylitis Ankylopoietica[Title/Abstract])) OR (Ankylosing Spondyloarthritis[Title/Abstract])) OR (Ankylosing Spondyloarthritides[Title/Abstract])) OR (Spondyloarthritides, Ankylosing[Title/Abstract])) OR (Spondyloarthritis, Ankylosing[Title/Abstract]))) OR (((((((((("Arthritis, Psoriatic"[Mesh]) OR (Psoriasis, Arthritic[Title/Abstract])) OR (Arthritic Psoriasis[Title/Abstract])) OR (Psoriatic Arthritis[Title/Abstract])) OR (Psoriasis Arthropathica[Title/Abstract])) OR (Psoriatic Arthropathy[Title/Abstract])) OR (Arthropathies, Psoriatic[Title/Abstract])) OR (Arthropathy, Psoriatic[Title/Abstract])) OR (Psoriatic Arthropathies[Title/Abstract])) OR (Arthritis, Psoriatic[Title/Abstract]))) |
| **Cochrane Central Register of Randomized Trials** | #1 (Adalimumab):ti,ab,kw OR (Humira):ti,ab,kw OR (D2E7 Antibody):ti,ab,kw OR (Antibody, D2E7):ti,ab,kw OR (Certolizumab pegol):ti,ab,kw (Word variations have been searched)  #2 (Cimzia):ti,ab,kw OR (CDP870):ti,ab,kw OR (CDP 870):ti,ab,kw OR (Etanercept):ti,ab,kw OR (TNFR-Fc Fusion Protein):ti,ab,kw  #3 (Fusion Protein, TNFR-Fc):ti,ab,kw OR (TNFR Fc Fusion Protein):ti,ab,kw OR (TNR 001):ti,ab,kw OR (TNT Receptor Fusion Protein):ti,ab,kw OR (TNR-001):ti,ab,kw  #4 (Golimumab):ti,ab,kw OR (CNTO-148):ti,ab,kw OR (CNTO 148):ti,ab,kw OR (Simponi):ti,ab,kw OR (Infliximab):ti,ab,kw  #5 (Monoclonal Antibody cA2):ti,ab,kw OR (cA2, Monoclonal Antibody):ti,ab,kw OR (MAb cA2):ti,ab,kw OR (Remicade):ti,ab,kw  #6 MeSH descriptor: [Adalimumab]  #7 MeSH descriptor: [Certolizumab Pegol]  #8 MeSH descriptor: [Etanercept]  #9 #1 OR #2 OR #3 OR #4 OR #5 OR #6 OR #7 OR #8  #10 MeSH descriptor: [Spondylitis, Ankylosing]  #11 MeSH descriptor: [Arthritis, Psoriatic]  #12 (Ankylosing Spondylitis):ti,ab,kw OR (Spondyloarthritis Ankylopoietica):ti,ab,kw OR (Ankylosing Spondylarthritis):ti,ab,kw OR (Ankylosing Spondylarthritides):ti,ab,kw OR (Spondylarthritides, Ankylosing):ti,ab,kw  #13 MeSH descriptor: [Arthritis, Rheumatoid]  #14 (Spondylarthritis, Ankylosing):ti,ab,kw OR (Ankylosing Spondylitis):ti,ab,kw OR (Spondylarthritis Ankylopoietica):ti,ab,kw OR (Bechterew Disease):ti,ab,kw OR (Bechterew's Disease):ti,ab,kw  #15 (Bechterews Disease):ti,ab,kw OR (Marie-Struempell Disease):ti,ab,kw OR (Marie Struempell Disease):ti,ab,kw OR (Rheumatoid Spondylitis):ti,ab,kw OR (Spondylitis, Rheumatoid):ti,ab,kw  #16 (Spondylitis Ankylopoietica):ti,ab,kw OR (Ankylosing Spondyloarthritis):ti,ab,kw OR (Ankylosing Spondyloarthritides):ti,ab,kw OR (Spondyloarthritides, Ankylosing):ti,ab,kw OR (Spondyloarthritis, Ankylosing):ti,ab,kw  #17 (Rheumatoid Arthritis):ti,ab,kw OR (Psoriasis, Arthritic):ti,ab,kw OR (Arthritic Psoriasis):ti,ab,kw OR (Psoriatic Arthritis):ti,ab,kw OR (Psoriasis Arthropathica):ti,ab,kw  #18 (Psoriatic Arthropathy):ti,ab,kw OR (Arthropathies, Psoriatic):ti,ab,kw OR (Arthropathy, Psoriatic):ti,ab,kw OR (Psoriatic Arthropathies):ti,ab,kw  #19 #10 OR #11 OR #12 OR #13 OR #14 OR #15 OR #16 OR #17 OR #18  #20 (infect*)  #21 (TNF-α inhibitor):ti,ab,kw OR (Tumor necrosis factor-α inhibitor):ti,ab,kw OR (anti-tumor necrosis factor-α):ti,ab,kw OR (antitumor necrosis factor):ti,ab,kw OR (anti-TNF-α):ti,ab,kw  #22 (TNF blocker):ti,ab,kw OR (TNFalpha inhibitor):ti,ab,kw OR (TNF-alpha inhibitor):ti,ab,kw OR (Tumor Necrosis Factor alpha inhibitor):ti,ab,kw OR (Anti-TNFalpha):ti,ab,kw  #23 (Anti-tumor necrosis factor-alpha):ti,ab,kw  #24 #21 OR #22 OR #23  #25 #9 OR #24  #26 #25 AND #19 AND #20 |
| **Embase** | #1 'adalimumab'/exp  #2 'certolizumab pegol'/exp  #3 'etanercept'/exp  #4 'golimumab'/exp  #5 'infliximab'/exp  #6 adalimumab:ti,ab,kw OR humira:ti,ab,kw OR 'd2e7 antibody':ti,ab,kw OR 'antibody, d2e7':ti,ab,kw OR 'certolizumab pegol':ti,ab,kw OR cimzia:ti,ab,kw OR cdp870:ti,ab,kw OR 'cdp 870':ti,ab,kw OR etanercept:ti,ab,kw OR 'tnfr-fc fusion protein':ti,ab,kw OR 'fusion protein, tnfr-fc':ti,ab,kw OR 'tnfr fc fusion protein':ti,ab,kw OR 'tnt receptor fusion protein':ti,ab,kw OR 'tnr 001':ti,ab,kw OR 'tnf receptor type ii-igg fusion protein':ti,ab,kw OR 'tnf receptor type ii igg fusion protein':ti,ab,kw OR 'recombinant human dimeric tnf receptor type ii-igg fusion protein':ti,ab,kw OR 'recombinant human dimeric tnf receptor type ii igg fusion protein':ti,ab,kw OR enbrel:ti,ab,kw OR golimumab:ti,ab,kw OR 'cnto 148':ti,ab,kw OR simponi:ti,ab,kw  #7 infliximab:ti,ab,kw OR 'monoclonal antibody ca2':ti,ab,kw OR 'ca2, monoclonal antibody':ti,ab,kw OR 'mab ca2':ti,ab,kw OR remicade:ti,ab,kw  #8 'tnf-α inhibitor':ti,ab,kw OR ('tumor necrosis factor-α':ti,ab,kw AND 'tnf α':ti,ab,kw AND inhibitor:ti,ab,kw) OR ('anti-tumor necrosis factor-α':ti,ab,kw AND tnfα:ti,ab,kw) OR ('antitumor necrosis factor':ti,ab,kw AND tnf:ti,ab,kw) OR 'anti tnf α':ti,ab,kw OR 'tnf blocker':ti,ab,kw OR 'tnfalpha inhibitor':ti,ab,kw OR 'tnf-alpha inhibitor':ti,ab,kw OR 'tumor necrosis factor alpha inhibitor':ti,ab,kw OR 'anti tnfalpha':ti,ab,kw OR 'anti-tumor necrosis factor-alpha':ti,ab,kw  #9 'rheumatoid arthritis'/exp  #10 'ankylosing spondylitis'/exp  #11 'psoriatic arthritis'/exp  #12 'rheumatoid arthritis':ti,ab,kw OR 'spondyloarthritis ankylopoietica':ti,ab,kw OR 'ankylosing spondylarthritis':ti,ab,kw OR 'ankylosing spondylarthritides':ti,ab,kw OR 'spondylarthritides, ankylosing':ti,ab,kw OR 'spondylarthritis, ankylosing':ti,ab,kw OR 'ankylosing spondylitis':ti,ab,kw OR 'spondylarthritis ankylopoietica':ti,ab,kw OR 'bechterew disease':ti,ab,kw OR 'bechterews disease':ti,ab,kw OR 'marie-struempell disease':ti,ab,kw OR 'marie struempell disease':ti,ab,kw OR 'rheumatoid spondylitis':ti,ab,kw OR 'spondylitis, rheumatoid':ti,ab,kw OR 'spondylitis ankylopoietica':ti,ab,kw OR 'ankylosing spondyloarthritis':ti,ab,kw OR 'ankylosing spondyloarthritides':ti,ab,kw OR 'spondyloarthritides, ankylosing':ti,ab,kw OR 'spondyloarthritis, ankylosing':ti,ab,kw OR 'arthritic psoriasis':ti,ab,kw OR 'psoriasis, arthritic':ti,ab,kw OR 'psoriatic arthritis':ti,ab,kw OR 'psoriasis arthropathica':ti,ab,kw OR 'psoriatic arthropathy':ti,ab,kw OR 'arthropathies, psoriatic':ti,ab,kw OR 'arthropathy, psoriatic':ti,ab,kw OR 'psoriatic arthropathies':ti,ab,kw  #13 infect*  #14 'controlled clinical trial':ti,ab,kw OR 'randomized controlled trial':ti,ab,kw OR rct:ti,ab,kw OR random*:ti,ab,kw OR trial:ti,ab,kw  #15 #1 OR #2 OR #3 OR #4 OR #5 OR #6 OR #7 OR #8  #16 #9 OR #10 OR #11 OR #12  #17 #13 AND #14 AND #15 AND #16 |

**Supplementary Appendix S1. References for included trials.**

[1] M.E. Weinblatt, J.M. Kremer, A.D. Bankhurst, K.J. Bulpitt, R.M. Fleischmann, R.I. Fox, C.G. Jackson, M. Lange, D.J. Burge, A trial of etanercept, a recombinant tumor necrosis factor receptor:Fc fusion protein, in patients with rheumatoid arthritis receiving methotrexate, N Engl J Med 340(4) (1999)253-9.

[2] P.E. Lipsky, D.M. van der Heijde, C.E. St, D.E. Furst, F.C. Breedveld, J.R. Kalden, J.S. Smolen, M. Weisman, P. Emery, M. Feldmann, G.R. Harriman, R.N. Maini, Infliximab and methotrexate in the treatment of rheumatoid arthritis. Anti-Tumor Necrosis Factor Trial in Rheumatoid Arthritis with Concomitant Therapy Study Group, N Engl J Med 343(22) (2000)1594-602.

[3] J. Braun, J. Brandt, J. Listing, A. Zink, R. Alten, W. Golder, E. Gromnica-Ihle, H. Kellner, A. Krause, M. Schneider, H. Sorensen, H. Zeidler, W. Thriene, J. Sieper, Treatment of active ankylosing spondylitis with infliximab: A randomised controlled multicentre trial, Lancet 359(9313) (2002)1187-93.

[4] J.D. Gorman, K.E. Sack, J.J. Davis, Treatment of ankylosing spondylitis by inhibition of tumor necrosis factor alpha, N Engl J Med 346(18) (2002)1349-56.

[5] J.J. Davis, D. Van Der Heijde, J. Braun, M. Dougados, J. Cush, D.O. Clegg, A. Kivitz, R. Fleischmann, R. Inman, W. Tsuji, Recombinant human tumor necrosis factor receptor (etanercept) for treating ankylosing spondylitis: A randomized, controlled trial, Arthritis Rheum 48(11) (2003)3230-6.

[6] D.E. Furst, M.H. Schiff, R.M. Fleischmann, V. Strand, C.A. Birbara, D. Compagnone, S.A. Fischkoff, E.K. Chartash, Adalimumab, a fully human anti tumor necrosis factor-alpha monoclonal antibody, and concomitant standard antirheumatic therapy for the treatment of rheumatoid arthritis: Results of STAR (Safety Trial of Adalimumab in Rheumatoid Arthritis), J Rheumatol 30(12) (2003)2563-71.

[7] E.C. Keystone, A.F. Kavanaugh, J.T. Sharp, H. Tannenbaum, Y. Hua, L.S. Teoh, S.A. Fischkoff, E.K. Chartash, Radiographic, clinical, and functional outcomes of treatment with adalimumab (a human anti-tumor necrosis factor monoclonal antibody) in patients with active rheumatoid arthritis receiving concomitant methotrexate therapy: A randomized, placebo-controlled, 52-week trial, Arthritis Rheum 50(5) (2004)1400-11.

[8] P.J. Mease, A.J. Kivitz, F.X. Burch, E.L. Siegel, S.B. Cohen, P. Ory, D. Salonen, J. Rubenstein, J.T. Sharp, W. Tsuji, Etanercept treatment of psoriatic arthritis: Safety, efficacy, and effect on disease progression, Arthritis Rheum 50(7) (2004)2264-72.

[9] C.E. St, D.M. van der Heijde, J.S. Smolen, R.N. Maini, J.M. Bathon, P. Emery, E. Keystone, M. Schiff, J.R. Kalden, B. Wang, K. Dewoody, R. Weiss, D. Baker, Combination of infliximab and methotrexate therapy for early rheumatoid arthritis: A randomized, controlled trial, Arthritis Rheum 50(11) (2004)3432-43.

[10] H. Marzo-Ortega, D. McGonagle, S. Jarrett, G. Haugeberg, E. Hensor, P. O'Connor, A.L. Tan, P.G. Conaghan, A. Greenstein, P. Emery, Infliximab in combination with methotrexate in active ankylosing spondylitis: A clinical and imaging study, Ann Rheum Dis 64(11) (2005)1568-75.

[11] P.J. Mease, D.D. Gladman, C.T. Ritchlin, E.M. Ruderman, S.D. Steinfeld, E.H. Choy, J.T. Sharp, P.A. Ory, R.J. Perdok, M.A. Weinberg, Adalimumab for the treatment of patients with moderately to severely active psoriatic arthritis: Results of a double-blind, randomized, placebo-controlled trial, Arthritis Rheum 52(10) (2005)3279-89.

[12] D. van der Heijde, B. Dijkmans, P. Geusens, J. Sieper, K. DeWoody, P. Williamson, J. Braun, Efficacy and safety of infliximab in patients with ankylosing spondylitis: Results of a randomized, placebo-controlled trial (ASSERT), Arthritis Rheum 52(2) (2005)582-91.

[13] T. Abe, T. Takeuchi, N. Miyasaka, H. Hashimoto, H. Kondo, Y. Ichikawa, I. Nagaya, A multicenter, double-blind, randomized, placebo controlled trial of infliximab combined with low dose methotrexate in Japanese patients with rheumatoid arthritis, J Rheumatol 33(1) (2006)37-44.

[14] D. van der Heijde, S.J. Da, M. Dougados, P. Geher, I. van der Horst-Bruinsma, X. Juanola, I. Olivieri, F. Raeman, L. Settas, J. Sieper, J. Szechinski, D. Walker, M.P. Boussuge, J.S. Wajdula, L. Paolozzi, S. Fatenejad, Etanercept 50 mg once weekly is as effective as 25 mg twice weekly in patients with ankylosing spondylitis, Ann Rheum Dis 65(12) (2006)1572-7.

[15] D. van der Heijde, A. Kivitz, M.H. Schiff, J. Sieper, B.A. Dijkmans, J. Braun, M. Dougados, J.D. Reveille, R.L. Wong, H. Kupper, J.J. Davis, Efficacy and safety of adalimumab in patients with ankylosing spondylitis: Results of a multicenter, randomized, double-blind, placebo-controlled trial, Arthritis Rheum 54(7) (2006)2136-46.

[16] R. Westhovens, D. Yocum, J. Han, A. Berman, I. Strusberg, P. Geusens, M.U. Rahman, The safety of infliximab, combined with background treatments, among patients with rheumatoid arthritis and various comorbidities: A large, randomized, placebo-controlled trial, Arthritis Rheum 54(4) (2006)1075-86.

[17] Y.H.F.H. Feng-Chun ZHANG, L.N.A.C. YAO, Infliximab versus placebo in rheumatoid arthritis patients receiving concomitant methotrexate: A preliminary study from China, APLAR Journal of Rheumatology (9) (2006)127–130.

[18] D. van der Heijde, L. Klareskog, R. Landewé, G.A.W. Bruyn, A. Cantagrel, P. Durez, G. Herrero-Beaumont, Y. Molad, C. Codreanu, G. Valentini, R. Zahora, R. Pedersen, D. MacPeek, J. Wajdula, S. Fatenejad, Disease remission and sustained halting of radiographic progression with combination etanercept and methotrexate in patients with rheumatoid arthritis, Arthritis & Rheumatism 56(12) (2007)3928-3939.

[19] M.C. Genovese, P.J. Mease, G.T. Thomson, A.J. Kivitz, R.J. Perdok, M.A. Weinberg, J. Medich, E.H. Sasso, Safety and efficacy of adalimumab in treatment of patients with psoriatic arthritis who had failed disease modifying antirheumatic drug therapy, J Rheumatol 34(5) (2007)1040-50.

[20] J. Kay, E.L. Matteson, B. Dasgupta, P. Nash, P. Durez, S. Hall, E.C. Hsia, J. Han, C. Wagner, Z. Xu, S. Visvanathan, M.U. Rahman, Golimumab in patients with active rheumatoid arthritis despite treatment with methotrexate: A randomized, double-blind, placebo-controlled, dose-ranging study, Arthritis & Rheumatism 58(4) (2008)964-975.

[21] R.D. Inman, J.C. Davis, D.V.D. Heijde, L. Diekman, J. Sieper, S.I. Kim, M. Mack, J. Han, S. Visvanathan, Z. Xu, B. Hsu, A. Beutler, J. Braun, Efficacy and safety of golimumab in patients with ankylosing spondylitis: Results of a randomized, double-blind, placebo-controlled, phase III trial, Arthritis & Rheumatism 58(11) (2008)3402-3412.

[22] V. Bejarano, M. Quinn, P.G. Conaghan, R. Reece, A.M. Keenan, D. Walker, A. Gough, M. Green, D. McGonagle, A. Adebajo, S. Jarrett, S. Doherty, L. Hordon, R. Melsom, K. Unnebrink, H. Kupper, P. Emery, Effect of the early use of the anti-tumor necrosis factor adalimumab on the prevention of job loss in patients with early rheumatoid arthritis, Arthritis Rheum 59(10) (2008)1467-74.

[23] E. Keystone, D. Heijde, D.J. Mason, R. Landewe, R.V. Vollenhoven, B. Combe, P. Emery, V. Strand, P. Mease, C. Desai, K. Pavelka, Certolizumab pegol plus methotrexate is significantly more effective than placebo plus methotrexate in active rheumatoid arthritis: Findings of a fifty-two-week, phase III, multicenter, randomized, double-blind, placebo-controlled, parallel-group study, Arthritis Rheum 58(11) (2008)3319-29.

[24] N. Miyasaka, Clinical investigation in highly disease-affected rheumatoid arthritis patients in Japan with adalimumab applying standard and general evaluation: The CHANGE study, Mod Rheumatol 18(3) (2008)252-62.

[25] P. Emery, F.C. Breedveld, S. Hall, P. Durez, D.J. Chang, D. Robertson, A. Singh, R.D. Pedersen, A.S. Koenig, B. Freundlich, Comparison of methotrexate monotherapy with a combination of methotrexate and etanercept in active, early, moderate to severe rheumatoid arthritis (COMET): A randomised, double-blind, parallel treatment trial, Lancet 372(9636) (2008)375-82.

[26] M. Schiff, M. Keiserman, C. Codding, S. Songcharoen, A. Berman, S. Nayiager, C. Saldate, T. Li, R. Aranda, J.C. Becker, C. Lin, P.L. Cornet, M. Dougados, Efficacy and safety of abatacept or infliximab vs placebo in ATTEST: A phase III, multi-centre, randomised, double-blind, placebo-controlled study in patients with rheumatoid arthritis and an inadequate response to methotrexate, Ann Rheum Dis 67(8) (2008)1096-103.

[27] D.Y. Chen, S.J. Chou, T.Y. Hsieh, Y.H. Chen, H.H. Chen, C.W. Hsieh, J.L. Lan, Randomized, double-blind, placebo-controlled, comparative study of human anti-TNF antibody adalimumab in combination with methotrexate and methotrexate alone in Taiwanese patients with active rheumatoid arthritis, J Formos Med Assoc 108(4) (2009)310-9.

[28] B. Combe, C. Codreanu, U. Fiocco, M. Gaubitz, P.P. Geusens, T.K. Kvien, K. Pavelka, P.N. Sambrook, J.S. Smolen, R. Khandker, A. Singh, J. Wajdula, S. Fatenejad, Efficacy, safety and patient-reported outcomes of combination etanercept and sulfasalazine versus etanercept alone in patients with rheumatoid arthritis: A double-blind randomised 2-year study, Ann Rheum Dis 68(7) (2009)1146-52.

[29] P. Emery, R.M. Fleischmann, L.W. Moreland, E.C. Hsia, I. Strusberg, P. Durez, P. Nash, E.J. Amante, M. Churchill, W. Park, B.A. Pons-Estel, M.K. Doyle, S. Visvanathan, W. Xu, M.U. Rahman, Golimumab, a human anti-tumor necrosis factor alpha monoclonal antibody, injected subcutaneously every four weeks in methotrexate-naive patients with active rheumatoid arthritis: Twenty-four-week results of a phase III, multicenter, randomized, double-blind, placebo-controlled study of golimumab before methotrexate as first-line therapy for early-onset rheumatoid arthritis, Arthritis Rheum 60(8) (2009)2272-83.

[30] R. Fleischmann, J. Vencovsky, R.F. van Vollenhoven, D. Borenstein, J. Box, G. Coteur, N. Goel, H.P. Brezinschek, A. Innes, V. Strand, Efficacy and safety of certolizumab pegol monotherapy every 4 weeks in patients with rheumatoid arthritis failing previous disease-modifying antirheumatic therapy: The FAST4WARD study, Ann Rheum Dis 68(6) (2009)805-11.

[31] A. Kavanaugh, I. McInnes, P. Mease, G.G. Krueger, D. Gladman, J. Gomez-Reino, K. Papp, J. Zrubek, S. Mudivarthy, M. Mack, S. Visvanathan, A. Beutler, Golimumab, a new human tumor necrosis factor alpha antibody, administered every four weeks as a subcutaneous injection in psoriatic arthritis: Twenty-four-week efficacy and safety results of a randomized, placebo-controlled study, Arthritis Rheum 60(4) (2009)976-86.

[32] E.C. Keystone, M.C. Genovese, L. Klareskog, E.C. Hsia, S.T. Hall, P.C. Miranda, J. Pazdur, S.C. Bae, W. Palmer, J. Zrubek, M. Wiekowski, S. Visvanathan, Z. Wu, M.U. Rahman, Golimumab, a human antibody to tumour necrosis factor {alpha} given by monthly subcutaneous injections, in active rheumatoid arthritis despite methotrexate therapy: The GO-FORWARD Study, Ann Rheum Dis 68(6) (2009)789-96.

[33] J. Smolen, R.B. Landewe, P. Mease, J. Brzezicki, D. Mason, K. Luijtens, R.F. van Vollenhoven, A. Kavanaugh, M. Schiff, G.R. Burmester, V. Strand, J. Vencovsky, D. van der Heijde, Efficacy and safety of certolizumab pegol plus methotrexate in active rheumatoid arthritis: The RAPID 2 study. A randomised controlled trial, Ann Rheum Dis 68(6) (2009)797-804.

[34] J.S. Smolen, J. Kay, M.K. Doyle, R. Landewe, E.L. Matteson, J. Wollenhaupt, N. Gaylis, F.T. Murphy, J.S. Neal, Y. Zhou, S. Visvanathan, E.C. Hsia, M.U. Rahman, Golimumab in patients with active rheumatoid arthritis after treatment with tumour necrosis factor alpha inhibitors (GO-AFTER study): A multicentre, randomised, double-blind, placebo-controlled, phase III trial, Lancet 374(9685) (2009)210-21.

[35] R.F. van Vollenhoven, N. Kinnman, E. Vincent, S. Wax, J. Bathon, Atacicept in patients with rheumatoid arthritis and an inadequate response to methotrexate: Results of a phase II, randomized, placebo-controlled trial, Arthritis Rheum 63(7) (2011)1782-92.

[36] A. Baranauskaite, H. Raffayova, N.V. Kungurov, A. Kubanova, A. Venalis, L. Helmle, S. Srinivasan, E. Nasonov, N. Vastesaeger, Infliximab plus methotrexate is superior to methotrexate alone in the treatment of psoriatic arthritis in methotrexate-naive patients: The RESPOND study, Ann Rheum Dis 71(4) (2012)541-8.

[37] E. Choy, F. McKenna, J. Vencovsky, R. Valente, N. Goel, B. Vanlunen, O. Davies, H.D. Stahl, R. Alten, Certolizumab pegol plus MTX administered every 4 weeks is effective in patients with RA who are partial responders to MTX, Rheumatology (Oxford) 51(7) (2012)1226-34.

[38] R. Fleischmann, M. Cutolo, M.C. Genovese, E.B. Lee, K.S. Kanik, S. Sadis, C.A. Connell, D. Gruben, S. Krishnaswami, G. Wallenstein, B.E. Wilkinson, S.H. Zwillich, Phase IIb dose-ranging study of the oral JAK inhibitor tofacitinib (CP-690,550) or adalimumab monotherapy versus placebo in patients with active rheumatoid arthritis with an inadequate response to disease-modifying antirheumatic drugs, Arthritis Rheum 64(3) (2012)617-29.

[39] Y. Tanaka, M. Harigai, T. Takeuchi, H. Yamanaka, N. Ishiguro, K. Yamamoto, N. Miyasaka, T. Koike, M. Kanazawa, T. Oba, T. Yoshinari, D. Baker, Golimumab in combination with methotrexate in Japanese patients with active rheumatoid arthritis: Results of the GO-FORTH study, Ann Rheum Dis 71(6) (2012)817-24.

[40] R.F. van Vollenhoven, R. Fleischmann, S. Cohen, E.B. Lee, M.J. Garcia, S. Wagner, S. Forejtova, S.H. Zwillich, D. Gruben, T. Koncz, G.V. Wallenstein, S. Krishnaswami, J.D. Bradley, B. Wilkinson, Tofacitinib or adalimumab versus placebo in rheumatoid arthritis, N Engl J Med 367(6) (2012)508-19.

[41] M.E. Weinblatt, R. Fleischmann, T.W. Huizinga, P. Emery, J. Pope, E.M. Massarotti, R.F. van Vollenhoven, J. Wollenhaupt, C.R. Bingham, B. Duncan, N. Goel, O.R. Davies, M. Dougados, Efficacy and safety of certolizumab pegol in a broad population of patients with active rheumatoid arthritis: Results from the REALISTIC phase IIIb study, Rheumatology (Oxford) 51(12) (2012)2204-14.

[42] J. Detert, H. Bastian, J. Listing, A. Weiss, S. Wassenberg, A. Liebhaber, K. Rockwitz, R. Alten, K. Kruger, R. Rau, C. Simon, E. Gremmelsbacher, T. Braun, B. Marsmann, V. Hohne-Zimmer, K. Egerer, F. Buttgereit, G.R. Burmester, Induction therapy with adalimumab plus methotrexate for 24 weeks followed by methotrexate monotherapy up to week 48 versus methotrexate therapy alone for DMARD-naive patients with early rheumatoid arthritis: HIT HARD, an investigator-initiated study, Ann Rheum Dis 72(6) (2013)844-50.

[43] A. Kavanaugh, R.M. Fleischmann, P. Emery, H. Kupper, L. Redden, B. Guerette, S. Santra, J.S. Smolen, Clinical, functional and radiographic consequences of achieving stable low disease activity and remission with adalimumab plus methotrexate or methotrexate alone in early rheumatoid arthritis: 26-Week results from the randomised, controlled OPTIMA study, Ann Rheum Dis 72(1) (2013)64-71.

[44] J.S. Smolen, P. Nash, P. Durez, S. Hall, E. Ilivanova, F. Irazoque-Palazuelos, P. Miranda, M.C. Park, K. Pavelka, R. Pedersen, A. Szumski, C. Hammond, A.S. Koenig, B. Vlahos, Maintenance, reduction, or withdrawal of etanercept after treatment with etanercept and methotrexate in patients with moderate rheumatoid arthritis (PRESERVE): A randomised controlled trial, Lancet 381(9870) (2013)918-29.

[45] T. Takeuchi, M. Harigai, Y. Tanaka, H. Yamanaka, N. Ishiguro, K. Yamamoto, N. Miyasaka, T. Koike, M. Kanazawa, T. Oba, T. Yoshinari, D. Baker, Golimumab monotherapy in Japanese patients with active rheumatoid arthritis despite prior treatment with disease-modifying antirheumatic drugs: Results of the phase 2/3, multicentre, randomised, double-blind, placebo-controlled GO-MONO study through 24 weeks, Ann Rheum Dis 72(9) (2013)1488-95.

[46] F. Huang, J. Gu, P. Zhu, C. Bao, J. Xu, H. Xu, H. Wu, G. Wang, Q. Shi, N. Andhivarothai, J. Anderson, A.L. Pangan, Efficacy and safety of adalimumab in Chinese adults with active ankylosing spondylitis: Results of a randomised, controlled trial, Ann Rheum Dis 73(3) (2014)587-94.

[47] T. Takeuchi, H. Yamanaka, N. Ishiguro, N. Miyasaka, M. Mukai, T. Matsubara, S. Uchida, H. Akama, H. Kupper, V. Arora, Y. Tanaka, Adalimumab, a human anti-TNF monoclonal antibody, outcome study for the prevention of joint damage in Japanese patients with early rheumatoid arthritis: The HOPEFUL 1 study, Ann Rheum Dis 73(3) (2014)536-43.

[48] K. Yamamoto, T. Takeuchi, H. Yamanaka, N. Ishiguro, Y. Tanaka, K. Eguchi, A. Watanabe, H. Origasa, K. Iwai, Y. Sakamaki, D. van der Heijde, N. Miyasaka, T. Koike, Efficacy and safety of certolizumab pegol without methotrexate co-administration in Japanese patients with active rheumatoid arthritis: The HIKARI randomized, placebo-controlled trial, Mod Rheumatol 24(4) (2014)552-60.

[49] K. Hobbs, A. Deodhar, B. Wang, B. Bitman, J. Nussbaum, J. Chung, D.H. Collier, Randomized, double-blind, placebo-controlled study to evaluate the efficacy and safety of etanercept in patients with moderately active rheumatoid arthritis despite DMARD therapy, Springerplus 4 (2015)113.

[50] J.S. Smolen, P. Emery, G.F. Ferraccioli, W. Samborski, F. Berenbaum, O.R. Davies, W. Koetse, O. Purcaru, B. Bennett, H. Burkhardt, Certolizumab pegol in rheumatoid arthritis patients with low to moderate activity: The CERTAIN double-blind, randomised, placebo-controlled trial, Ann Rheum Dis 74(5) (2015)843-50.

[51] Z. Li, F. Zhang, J. Kay, K. Fei, C. Han, Y. Zhuang, Z. Wu, E.C. Hsia, Efficacy and safety results from a Phase 3, randomized, placebo-controlled trial of subcutaneous golimumab in Chinese patients with active rheumatoid arthritis despite methotrexate therapy, Int J Rheum Dis 19(11) (2016)1143-1156.

[52] J.S. Smolen, G.R. Burmester, B. Combe, J.R. Curtis, S. Hall, B. Haraoui, R. van Vollenhoven, C. Cioffi, C. Ecoffet, L. Gervitz, L. Ionescu, L. Peterson, R. Fleischmann, Head-to-head comparison of certolizumab pegol versus adalimumab in rheumatoid arthritis: 2-Year efficacy and safety results from the randomised EXXELERATE study, Lancet 388(10061) (2016)2763-2774.

[53] P.C. Taylor, E.C. Keystone, D. van der Heijde, M.E. Weinblatt, C.M.L. Del, G.J. Reyes, S. Yakushin, T. Ishii, K. Emoto, S. Beattie, V. Arora, C. Gaich, T. Rooney, D. Schlichting, W.L. Macias, S. de Bono, Y. Tanaka, Baricitinib versus Placebo or Adalimumab in Rheumatoid Arthritis, N Engl J Med 376(7) (2017)652-662.

[54] Y.M. Kang, Y.E. Park, W. Park, J.Y. Choe, C.S. Cho, S.C. Shim, S.C. Bae, C.H. Suh, H.S. Cha, E.M. Koh, Y.W. Song, B. Yoo, S.S. Lee, M.C. Park, S.H. Lee, C. Arendt, W. Koetse, S.K. Lee, Rapid onset of efficacy predicts response to therapy with certolizumab plus methotrexate in patients with active rheumatoid arthritis, Korean J Intern Med 33(6) (2018)1224-1233.

[55] D. van der Heijde, W.J. Cheng-Chung, M. Dougados, P. Mease, A. Deodhar, W.P. Maksymowych, F. Van den Bosch, J. Sieper, T. Tomita, R. Landewe, F. Zhao, E. Krishnan, D.H. Adams, B. Pangallo, H. Carlier, Ixekizumab, an interleukin-17A antagonist in the treatment of ankylosing spondylitis or radiographic axial spondyloarthritis in patients previously untreated with biological disease-modifying anti-rheumatic drugs (COAST-V): 16 week results of a phase 3 randomised, double-blind, active-controlled and placebo-controlled trial, Lancet 392(10163) (2018)2441-2451.

[56] L. Bi, Y. Li, L. He, H. Xu, Z. Jiang, Y. Wang, X. Li, W. Wei, J. Gu, G. Wang, Z. Zhang, B. Zhou, Y. Liu, Z. Wu, H. Liu, D. He, Z. Lv, Z. Li, X. Zuo, L. Dong, H. Wu, H. Zhang, H. Chen, C. Bao, Z. Zhang, M. Zhang, H. Song, Y. Zheng, L. Jiang, X. Liu, M. Boehnlein, J. Dunkel, J. Shao, K. Harris, Z. Li, Efficacy and safety of certolizumab pegol in combination with methotrexate in methotrexate-inadequate responder Chinese patients with active rheumatoid arthritis: 24-Week results from a randomised, double-blind, placebo-controlled phase 3 study, Clin Exp Rheumatol 37(2) (2019)227-234.

[57] R. Fleischmann, A.L. Pangan, I.H. Song, E. Mysler, L. Bessette, C. Peterfy, P. Durez, A.J. Ostor, Y. Li, Y. Zhou, A.A. Othman, M.C. Genovese, Upadacitinib versus placebo or adalimumab in patients with rheumatoid arthritis and an inadequate response to methotrexate: Results of a phase III, Double-Blind, randomized controlled trial, Arthritis Rheumatol 71(11) (2019)1788-1800.

[58] S. Cohen, K. Tuckwell, T.R. Katsumoto, R. Zhao, J. Galanter, C. Lee, J. Rae, B. Toth, N. Ramamoorthi, J.A. Hackney, A. Berman, N. Damjanov, D. Fedkov, S. Jeka, L.W. Chinn, M.J. Townsend, A.M. Morimoto, M.C. Genovese, Fenebrutinib versus Placebo or Adalimumab in Rheumatoid Arthritis: A Randomized, Double-Blind, Phase II Trial (ANDES Study), Arthritis Rheumatol (2020).

[59] I.B. McInnes, J.K. Anderson, M. Magrey, J.F. Merola, Y. Liu, M. Kishimoto, S. Jeka, C. Pacheco-Tena, X. Wang, L. Chen, P. Zueger, J. Liu, A.L. Pangan, F. Behrens, Trial of upadacitinib and adalimumab for psoriatic arthritis, N Engl J Med 384(13) (2021)1227-1239.

[60] J.S. Smolen, E. Feist, S. Fatenejad, S.A. Grishin, E.V. Korneva, E.L. Nasonov, M.Y. Samsonov, R.M. Fleischmann, Olokizumab versus placebo or adalimumab in rheumatoid arthritis, N Engl J Med 387(8) (2022)715-726.

[61] I.B. McInnes, A. Asahina, L.C. Coates, R. Landewe, J.F. Merola, C.T. Ritchlin, Y. Tanaka, L. Gossec, A.B. Gottlieb, R.B. Warren, B. Ink, D. Assudani, R. Bajracharya, V. Shende, J. Coarse, P.J. Mease, Bimekizumab in patients with psoriatic arthritis, naive to biologic treatment: a randomised, double-blind, placebo-controlled, phase 3 trial (be optimal), Lancet 401(10370) (2023)25-37.

**Supplementary Figure S1.** **Risk of bias assessments for each study based on adjusted Cochrane risk of bias tool.**


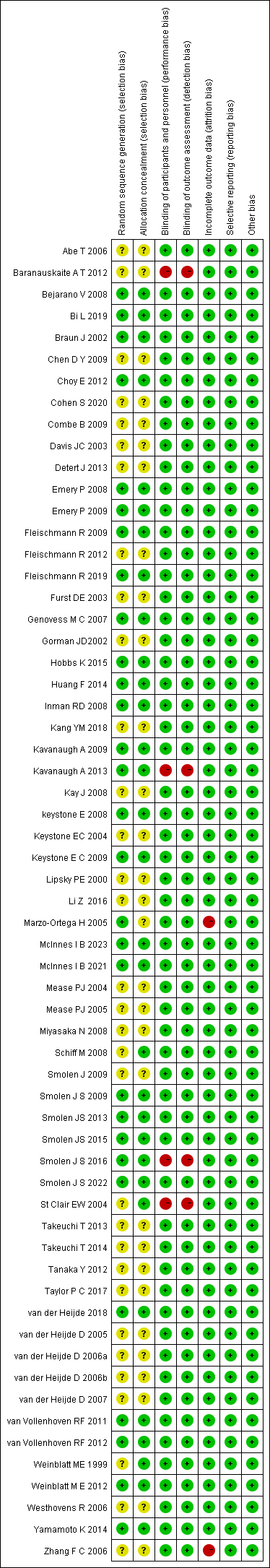

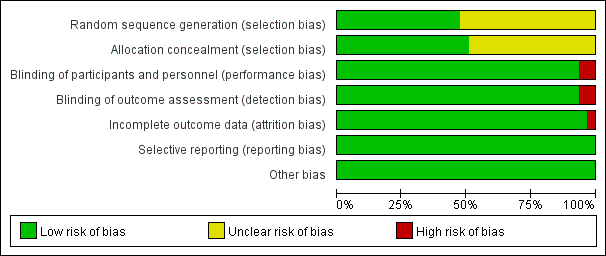


**Supplementary Figure S2. Pairwise meta-analysis of the effect of tumor necrosis factor-α inhibitors on the risk of infection. (A) Serious infection. (B) Any infection. (C) Opportunistic infection. (D)** **Herpes zoster. (E) Tuberculosis.**

**A**


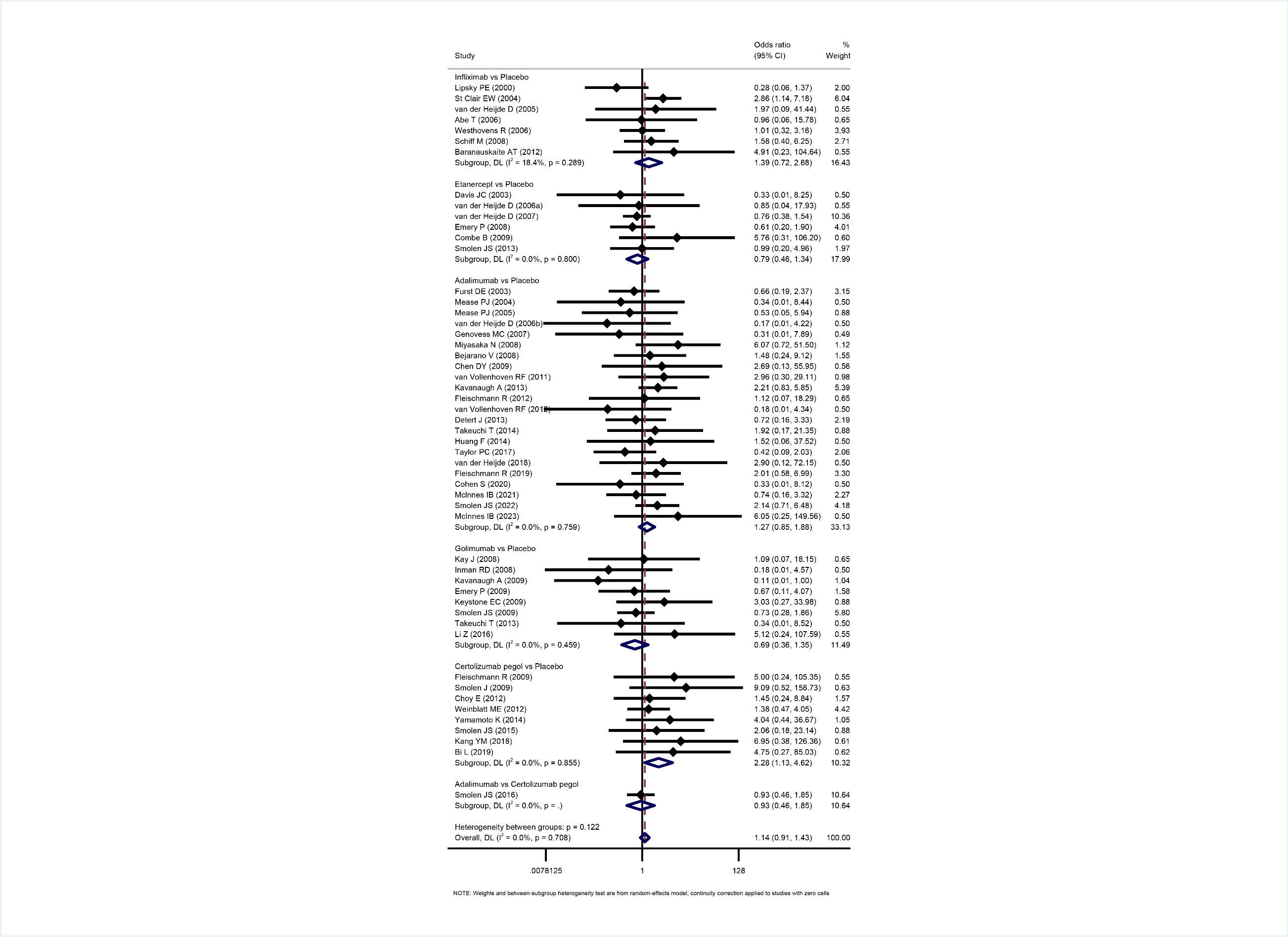


**B**


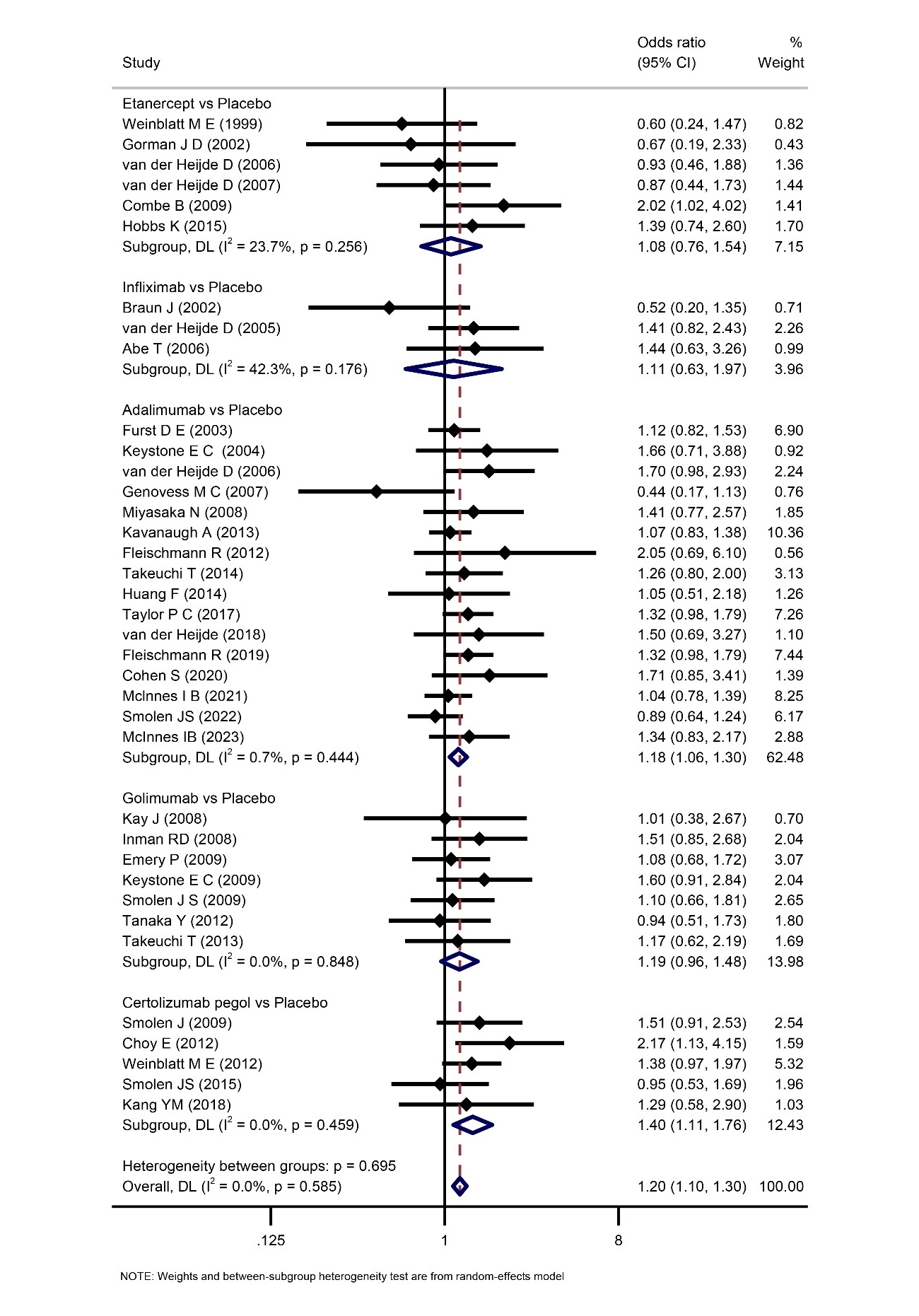


**C**


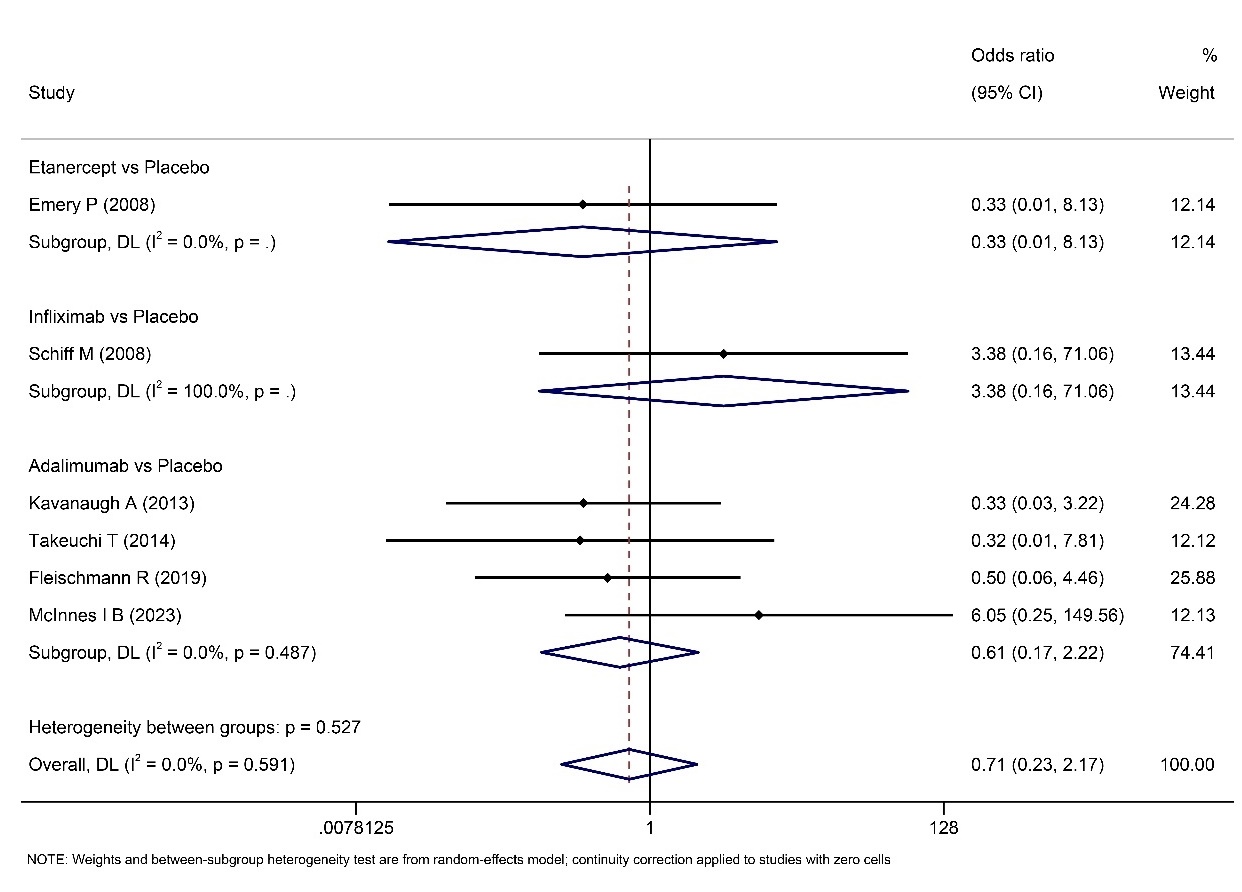


**D**


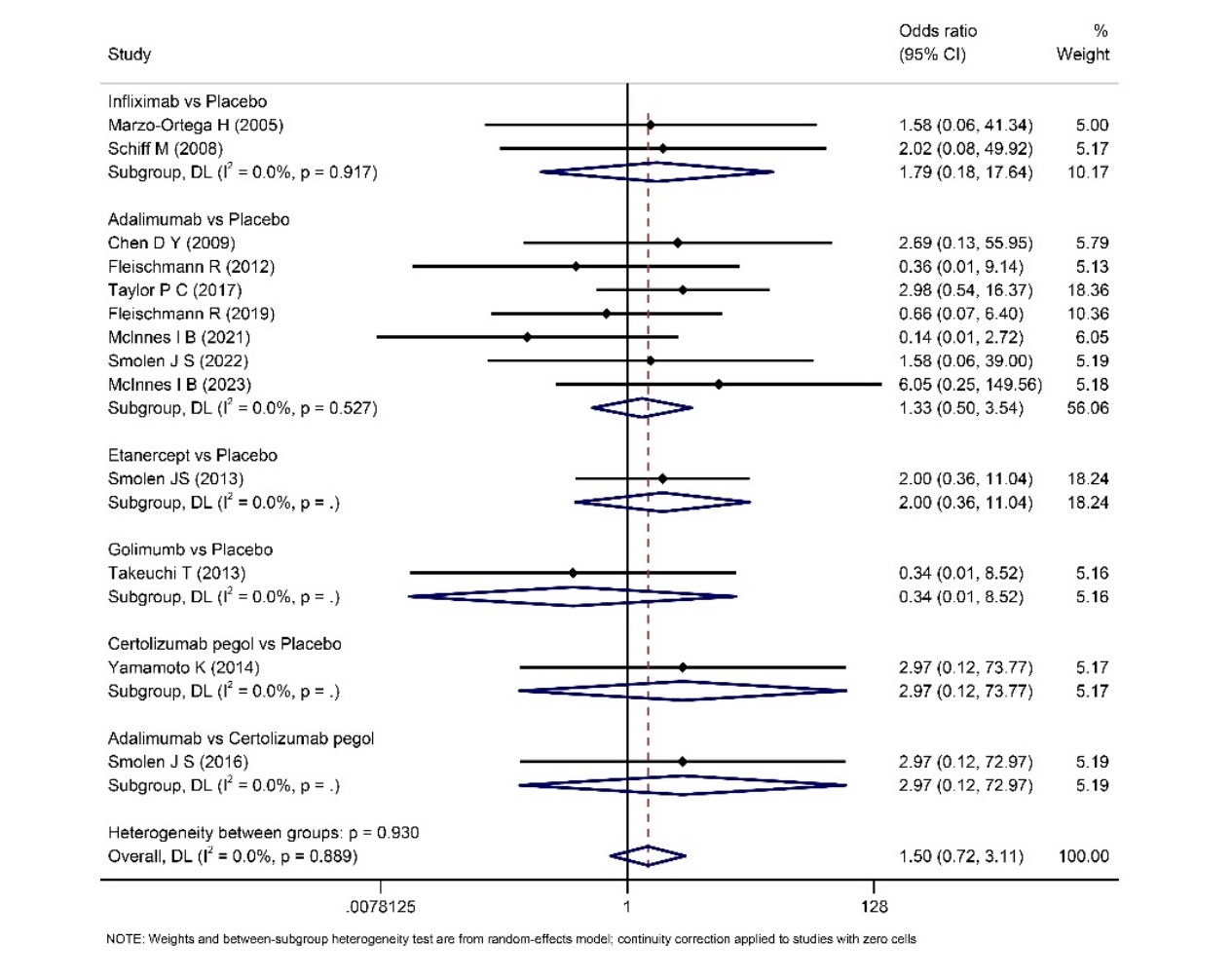


**E**


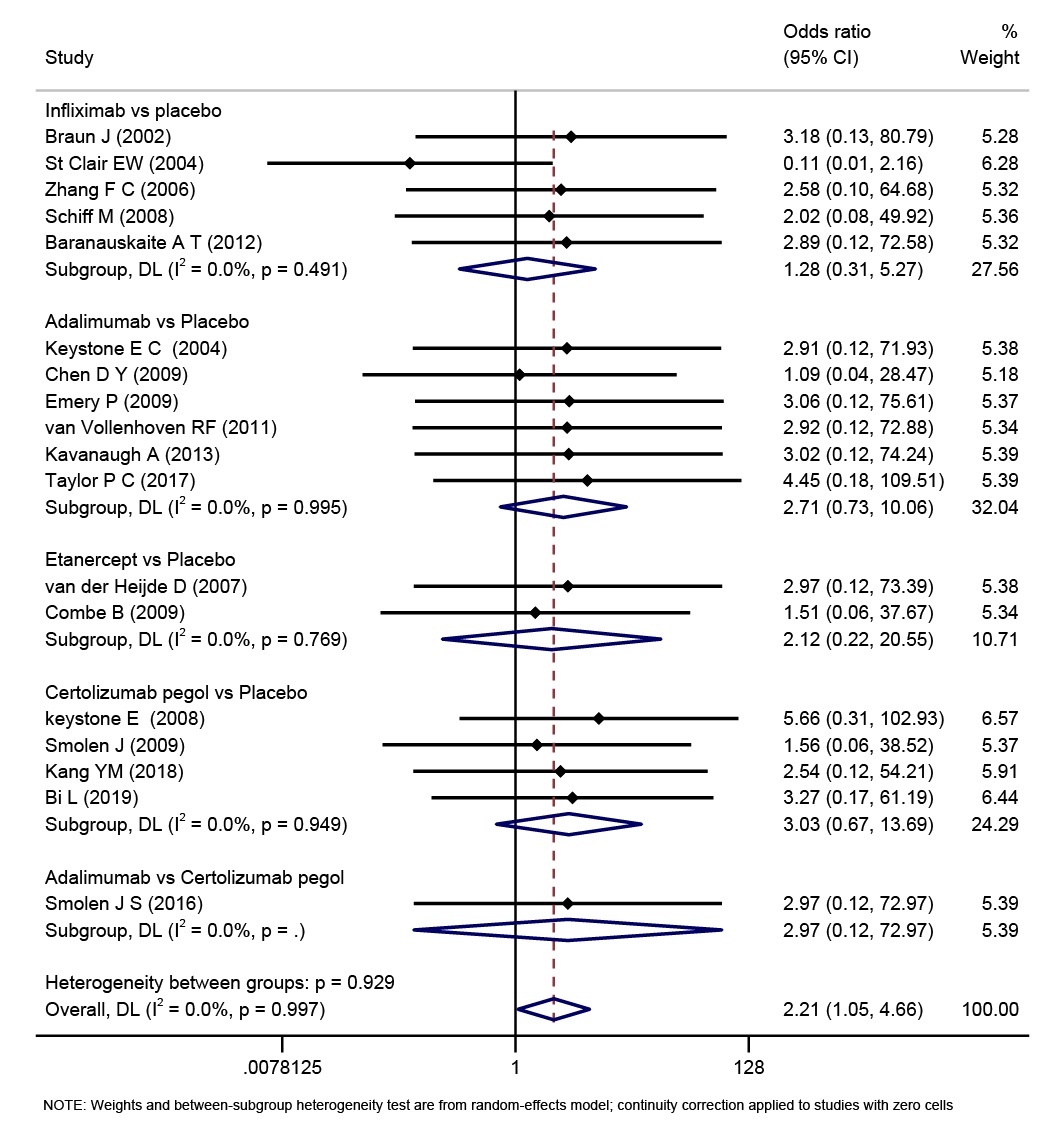


**Supplementary Figure S3. The publication bias assessment using Comparison-adjusted funnel-plot and funnel-plot. Abbreviation: 01, adalimumab; 02, golimumab; 03, certolizumab pegol; 04, infliximab; 05, etanercept; 06, placebo.** **(A) Serious infection. (B) Any infection. (C) Opportunistic infection. (D) Herpes zoster. (E) Tuberculosis.**

**A**


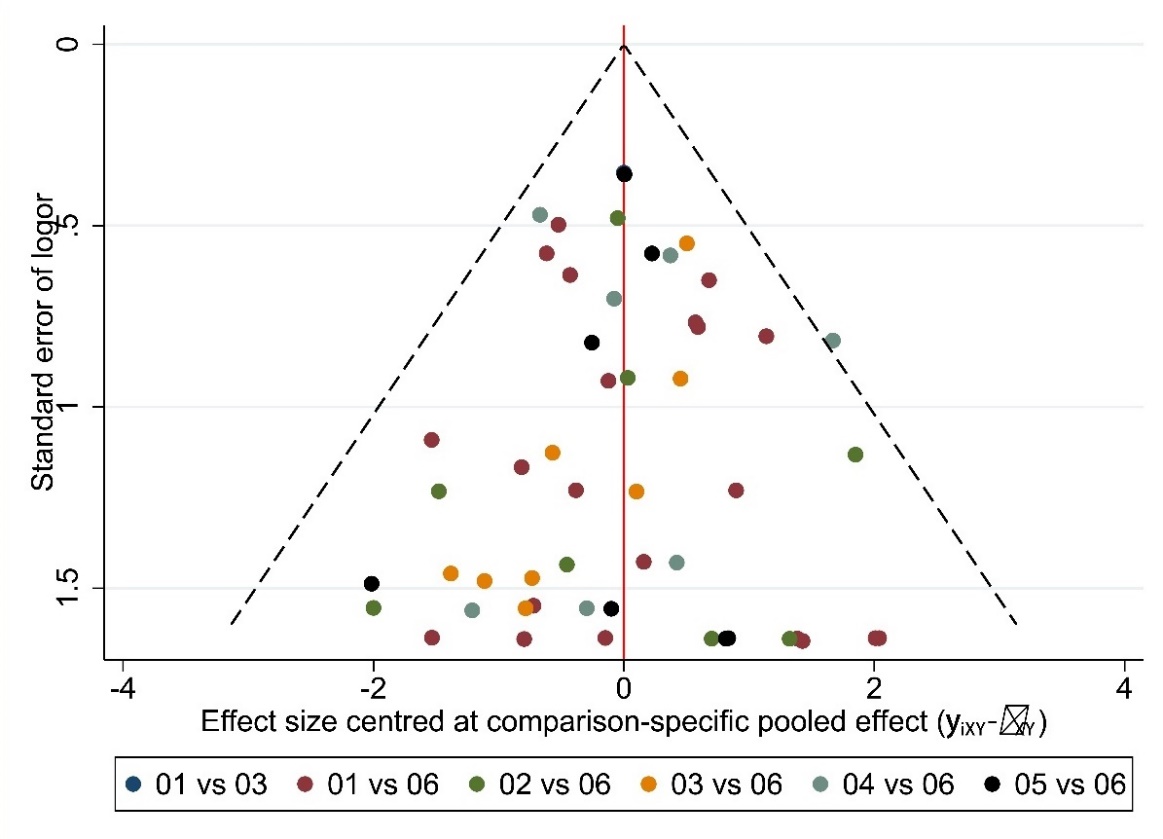


**B**


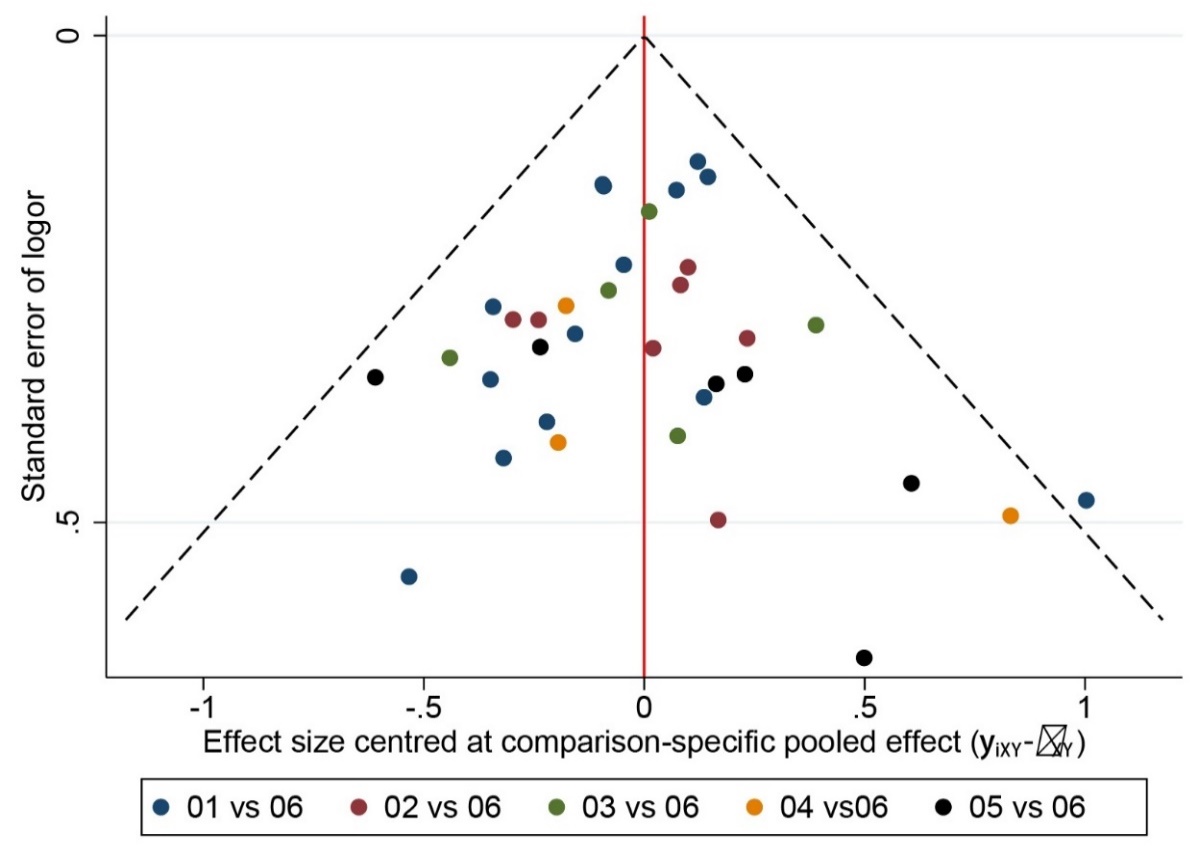


**C**


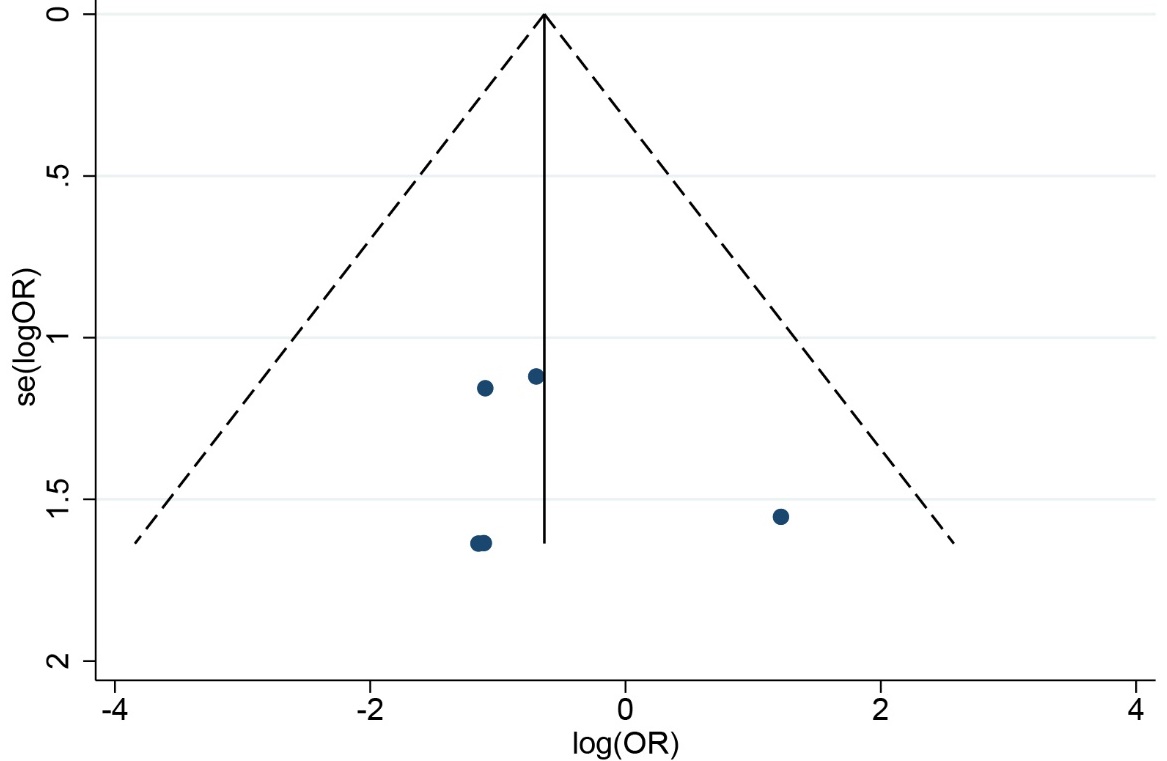


**D**


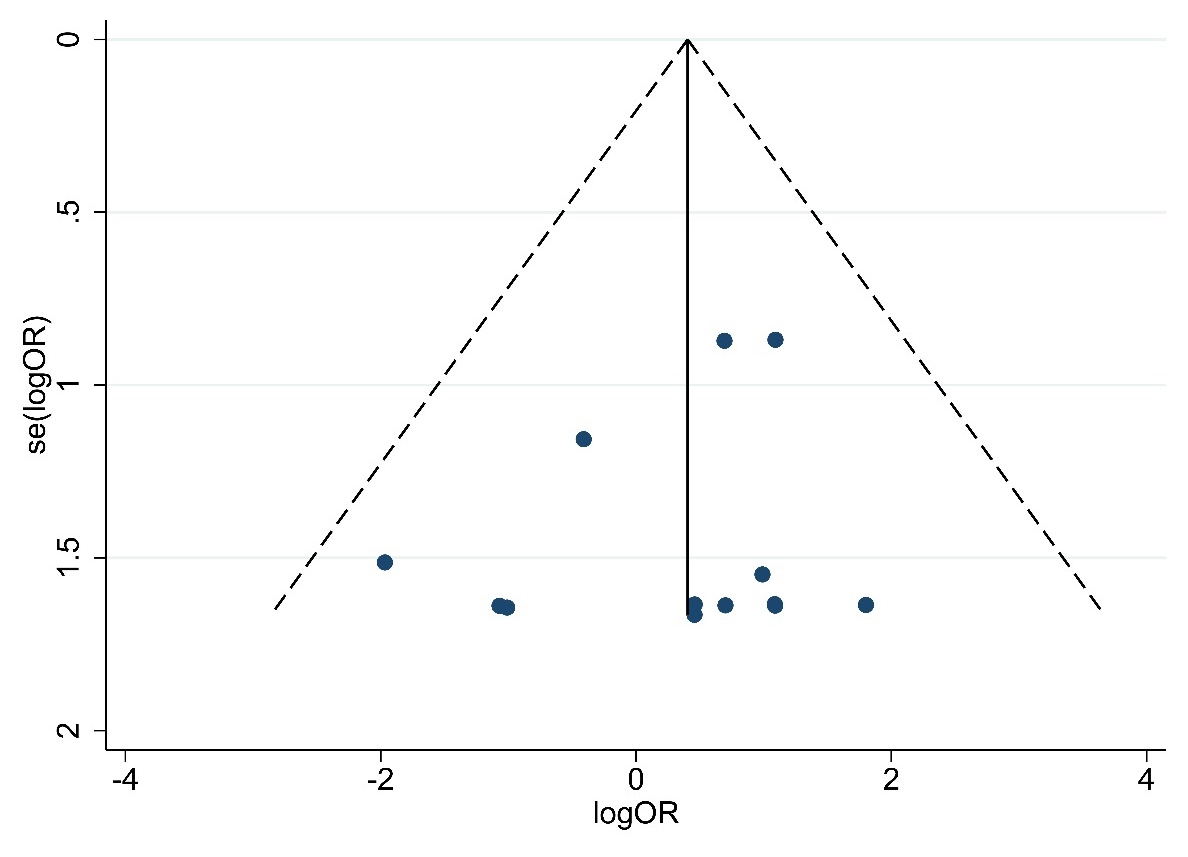


**E**


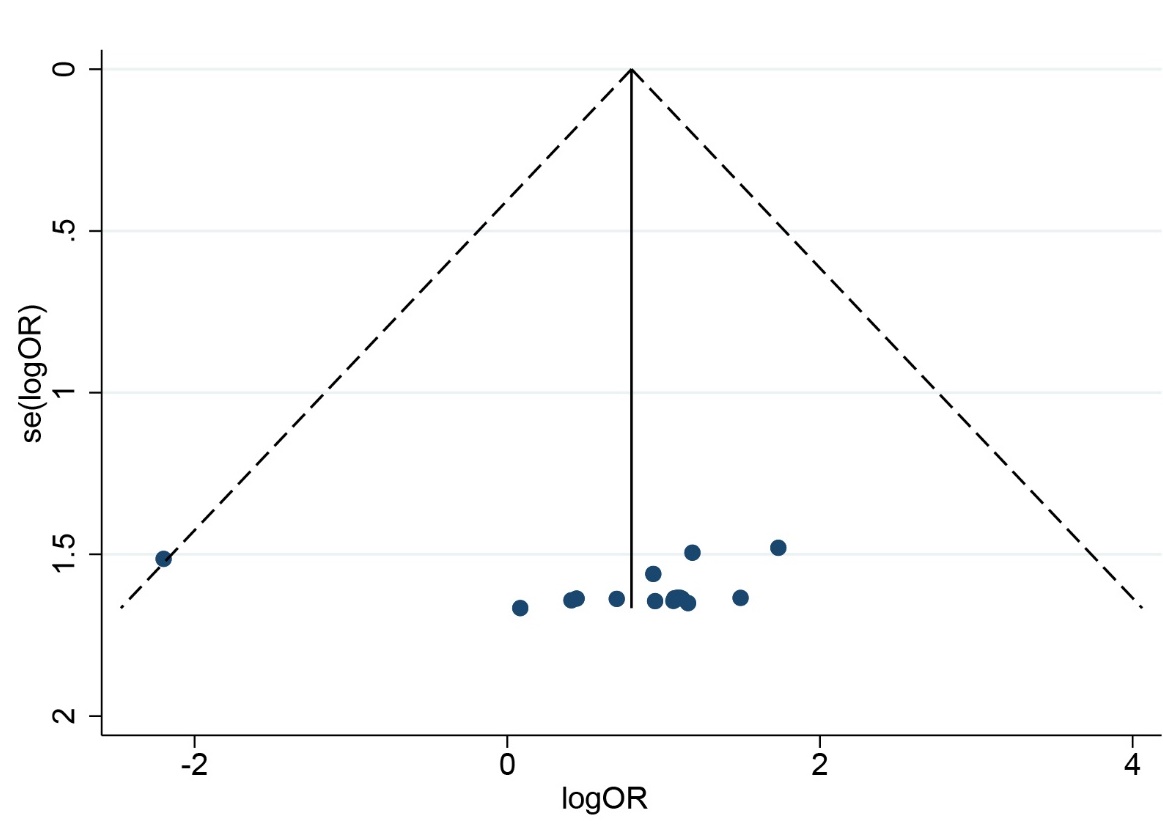


**Supplementary Figure S4. Sensitivity analysis by excluding trials with follow-up ⩾52weeks and sample size <50.**

**S4.1 Excluding trials with follow-up ⩾52weeks. (A) Serious infection. (B) Any infection. (C) Opportunistic infection. (D) Herpes zoster. (E) Tuberculosis.**

**A**


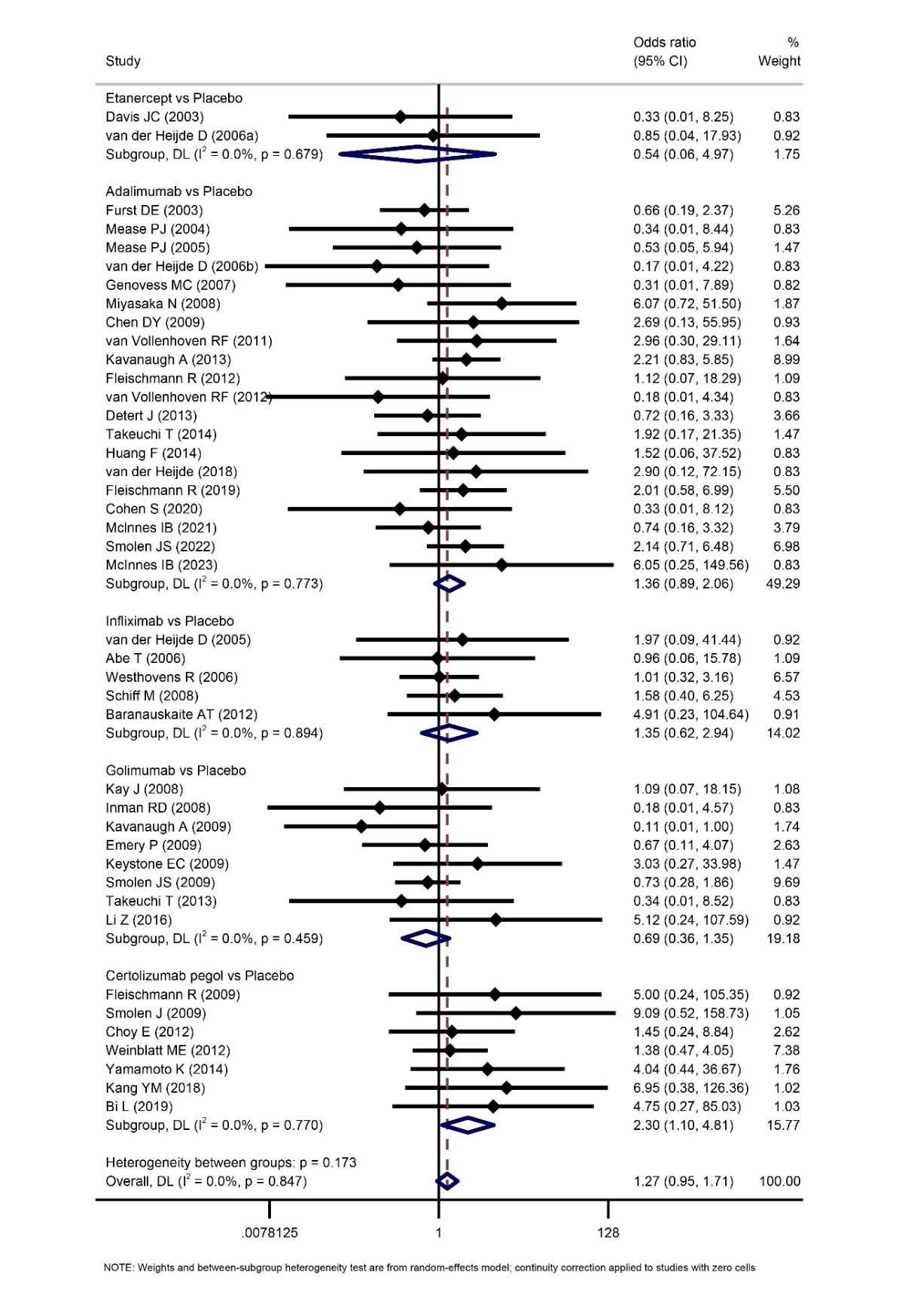


**B**


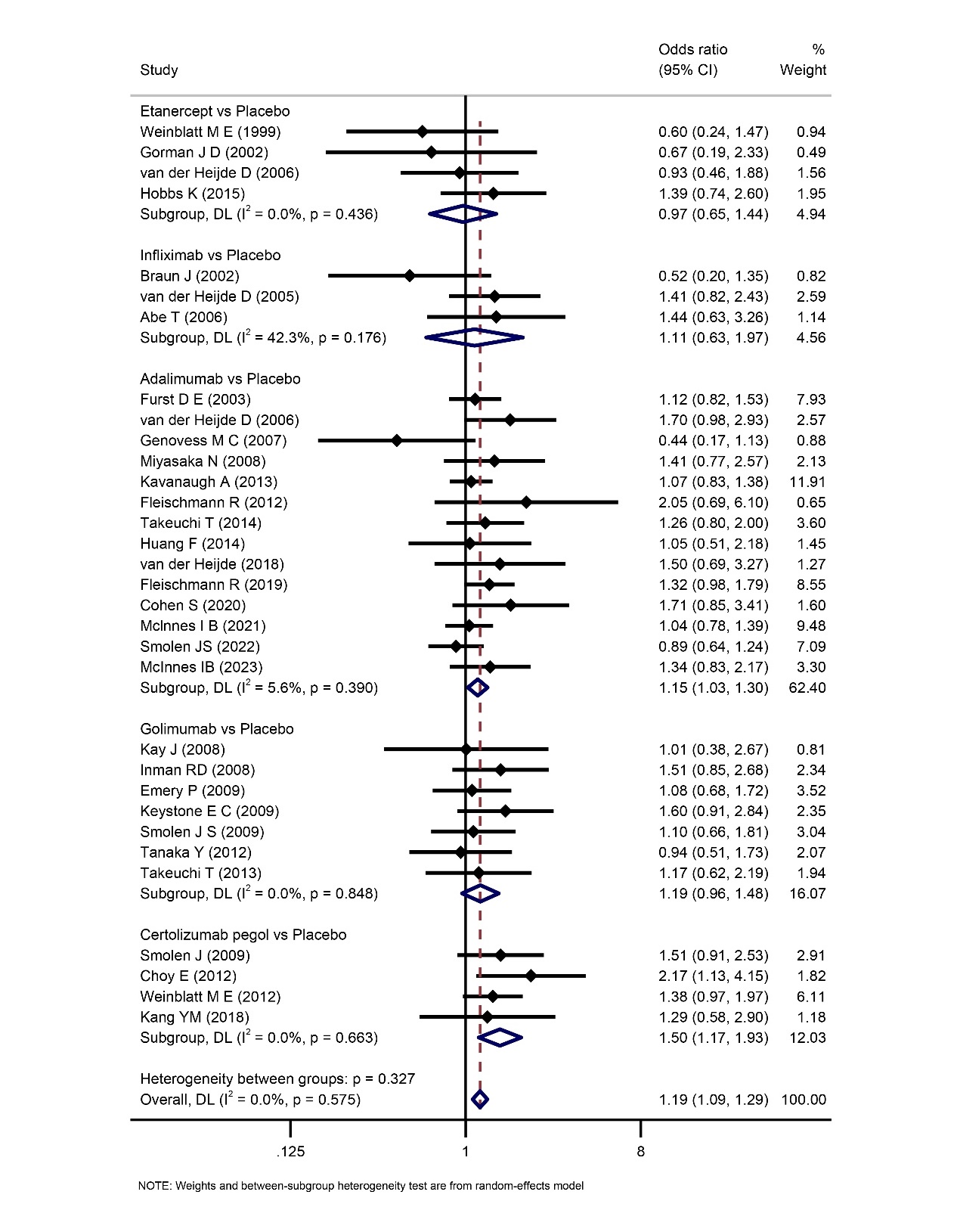


**C**


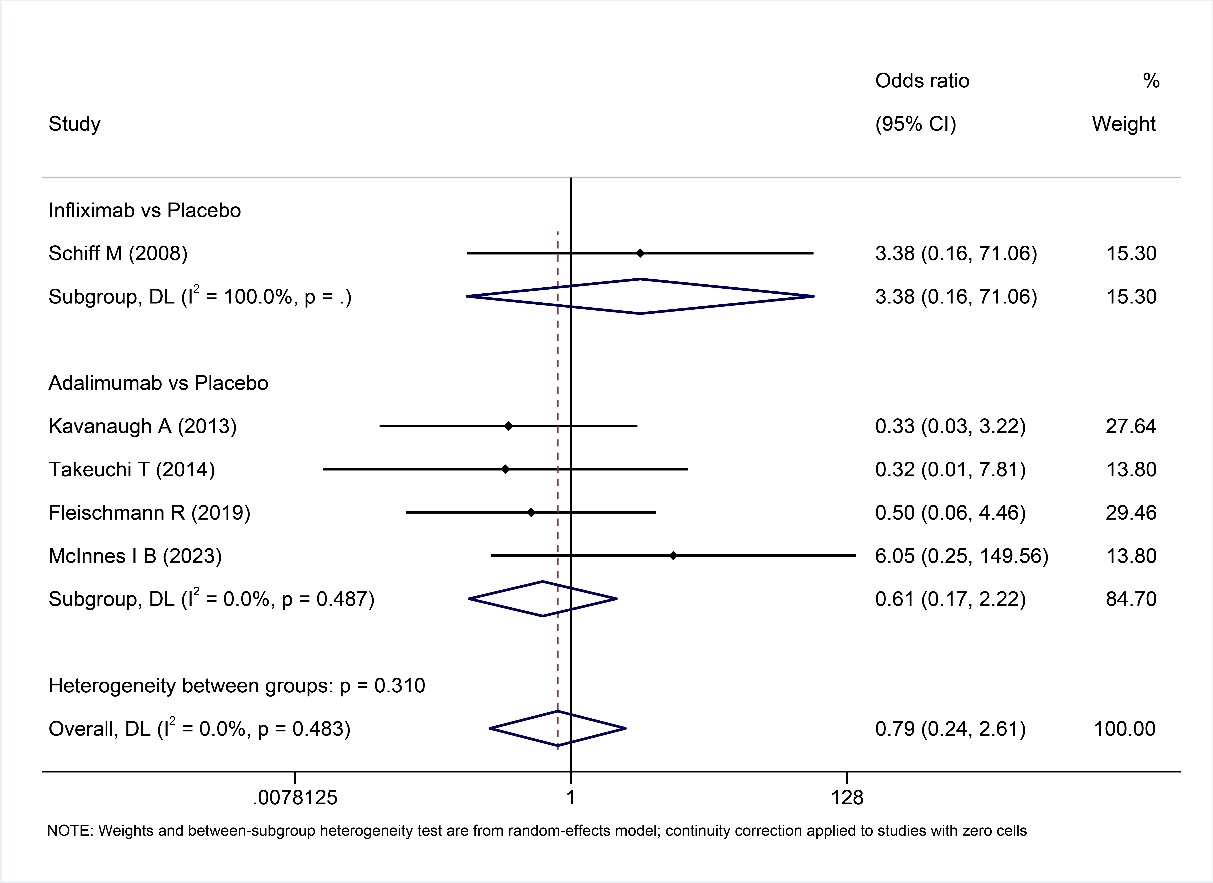


**D**


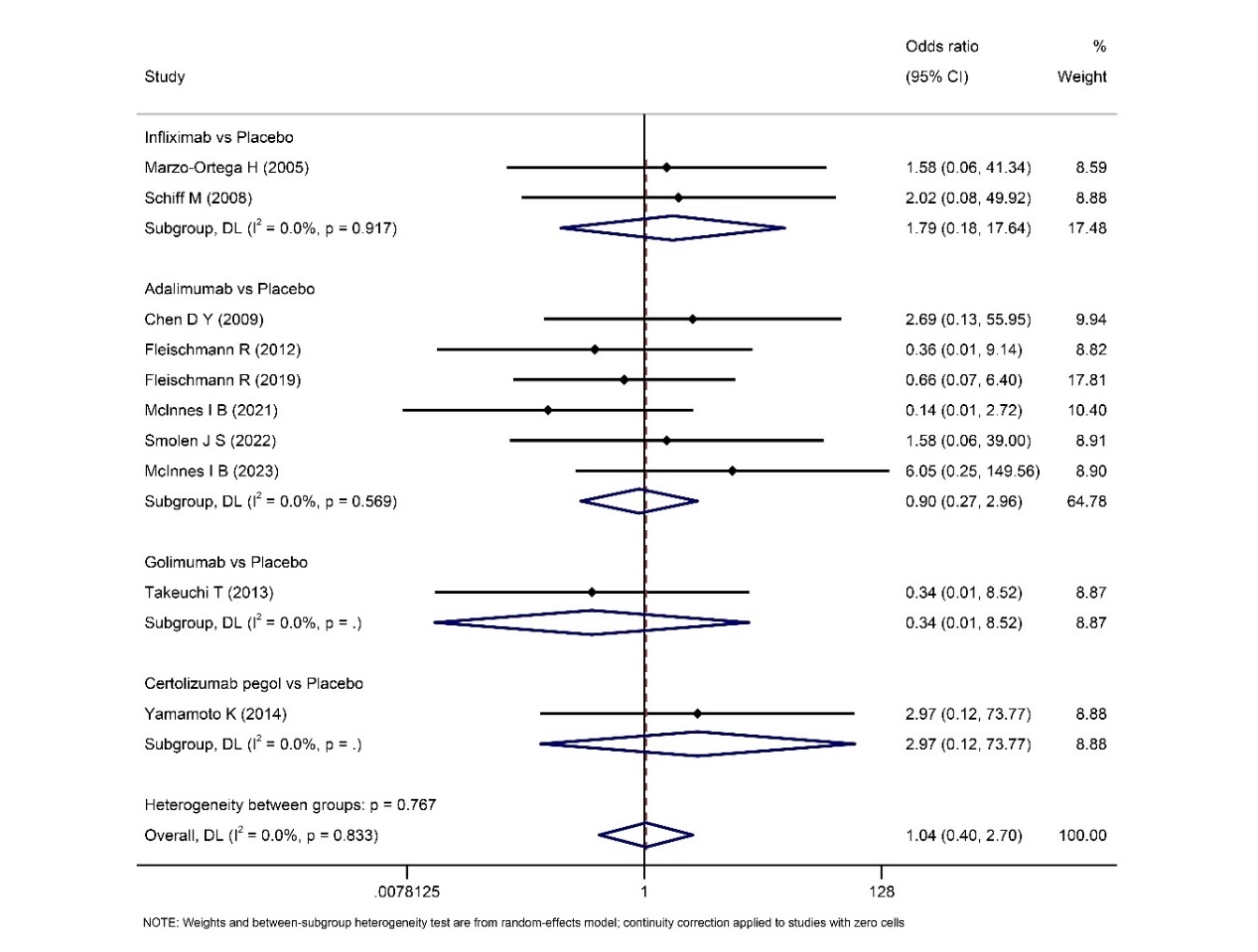


**E**

**
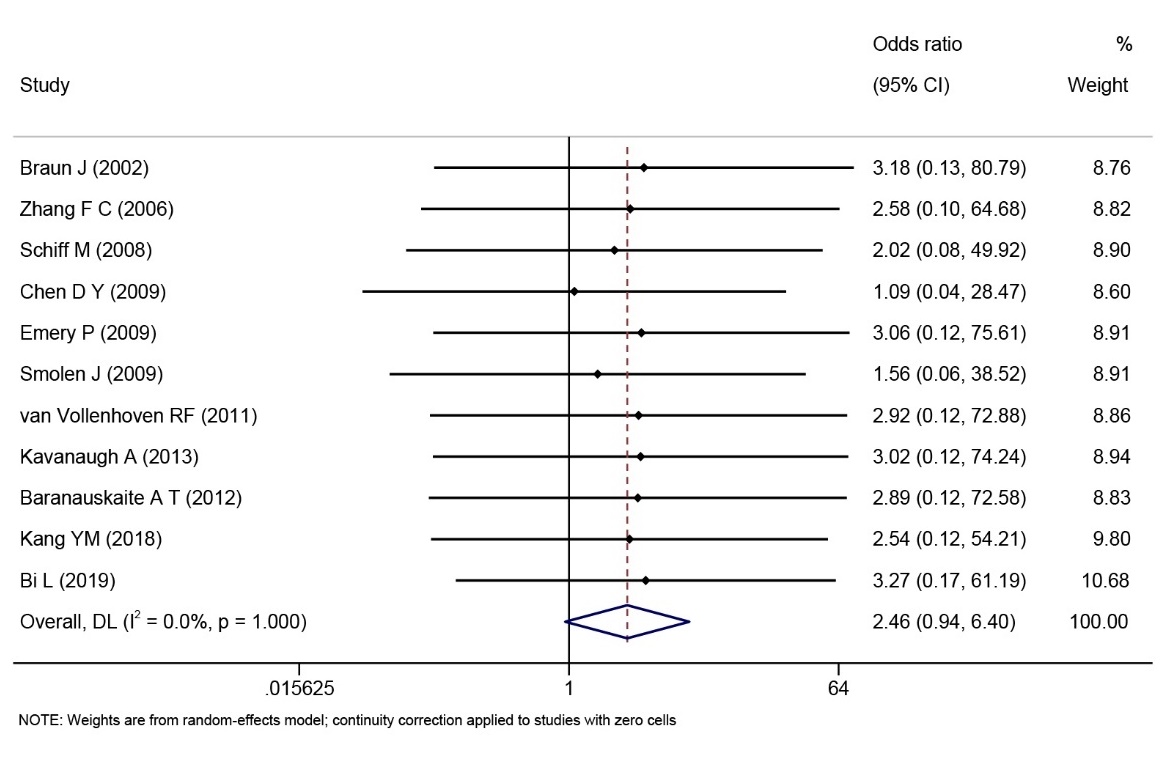
**

**S4.2 Excluding trials with sample size <50. (A) Serious infection. (B) Any infection. (C) Herpes zoster. (D) Tuberculosis.**

**A**


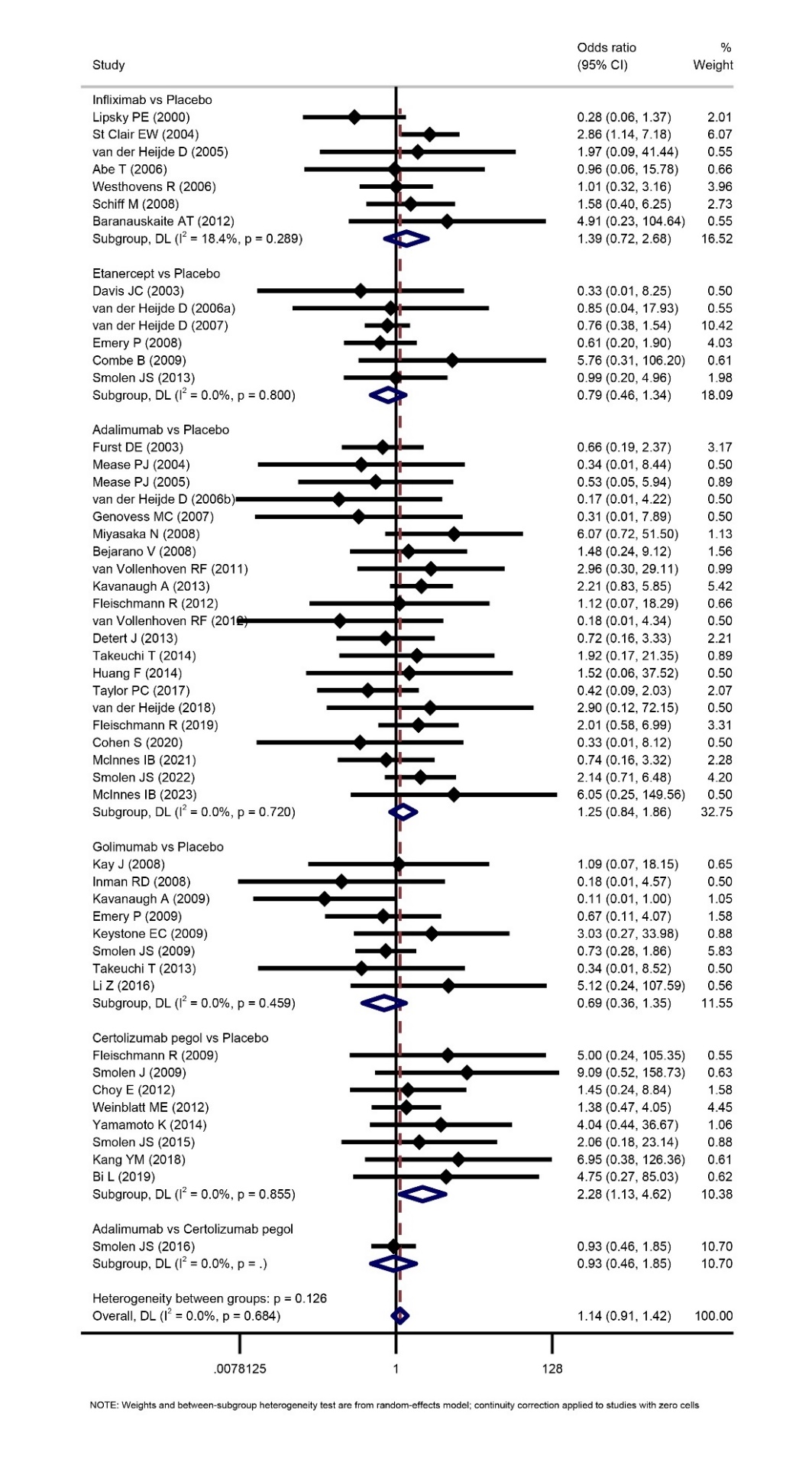


**B**


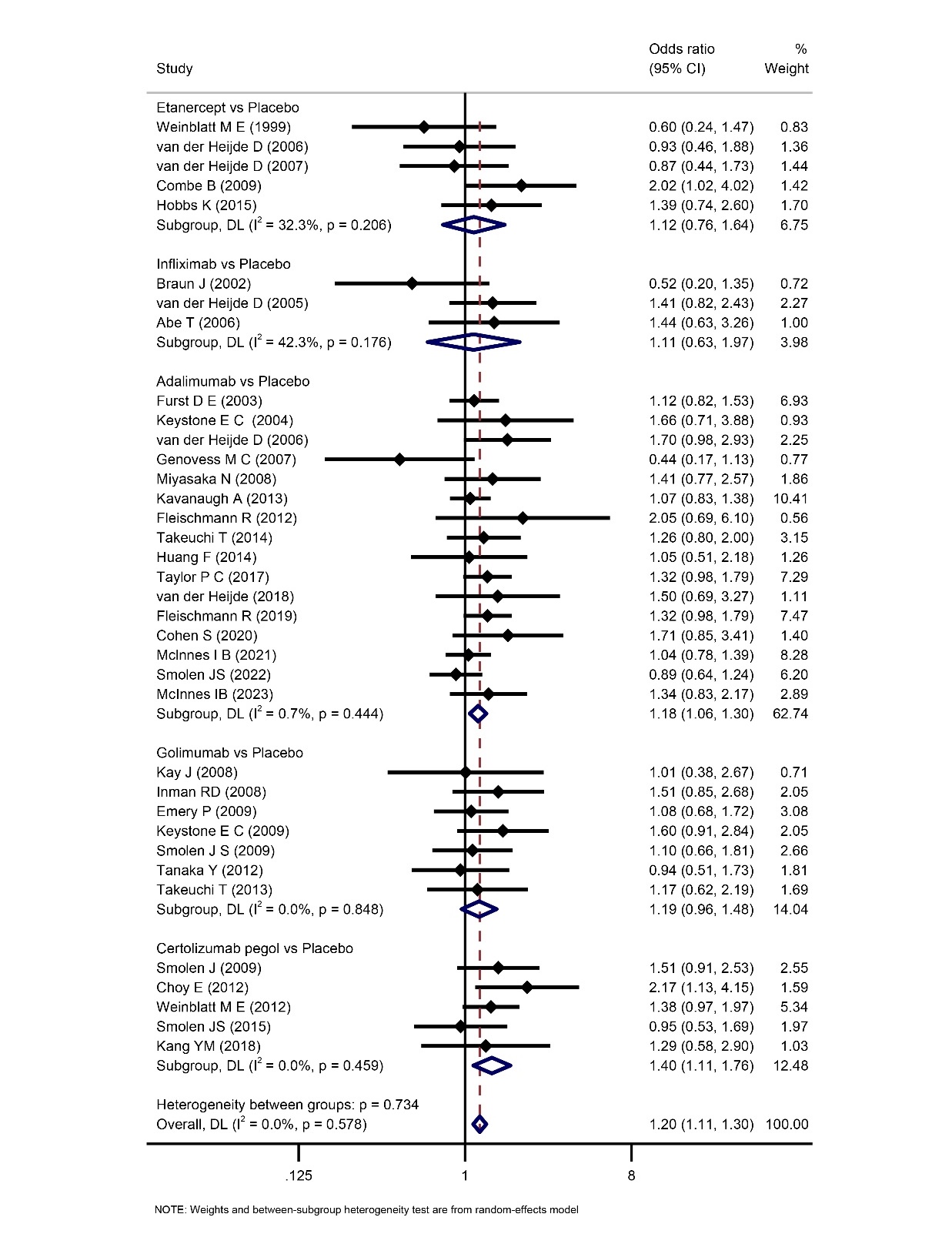


**C**

**D
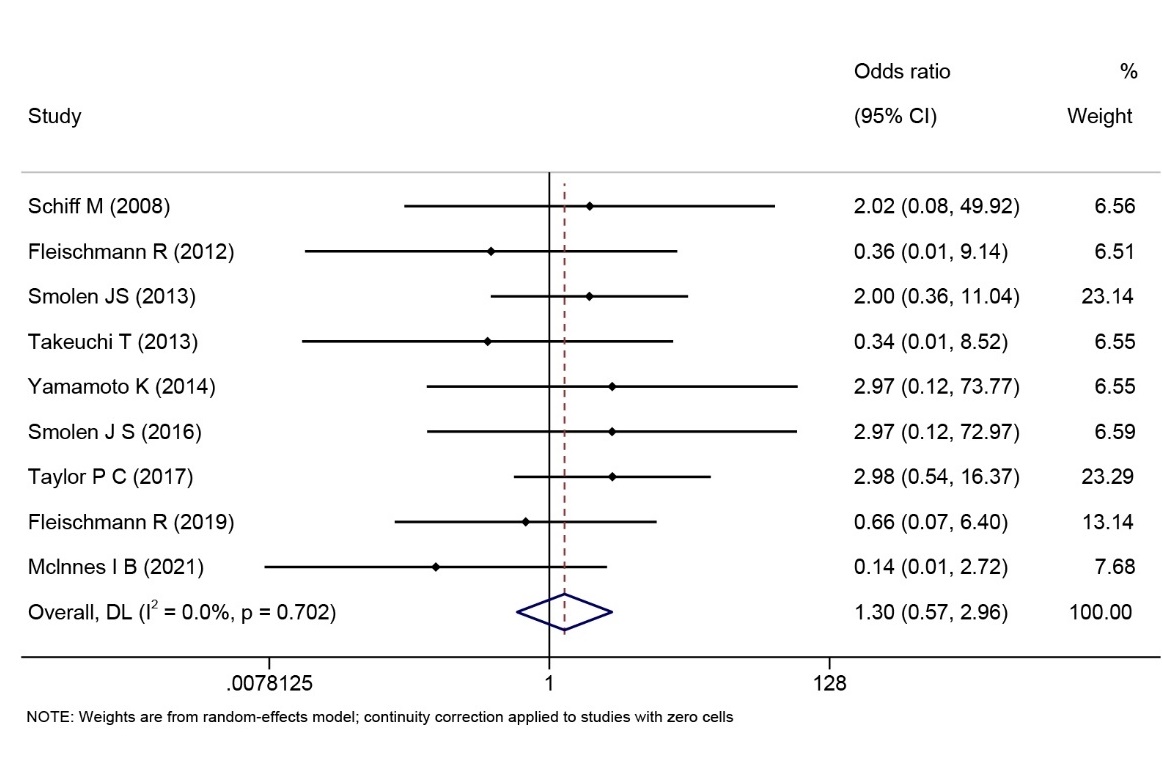
**

**
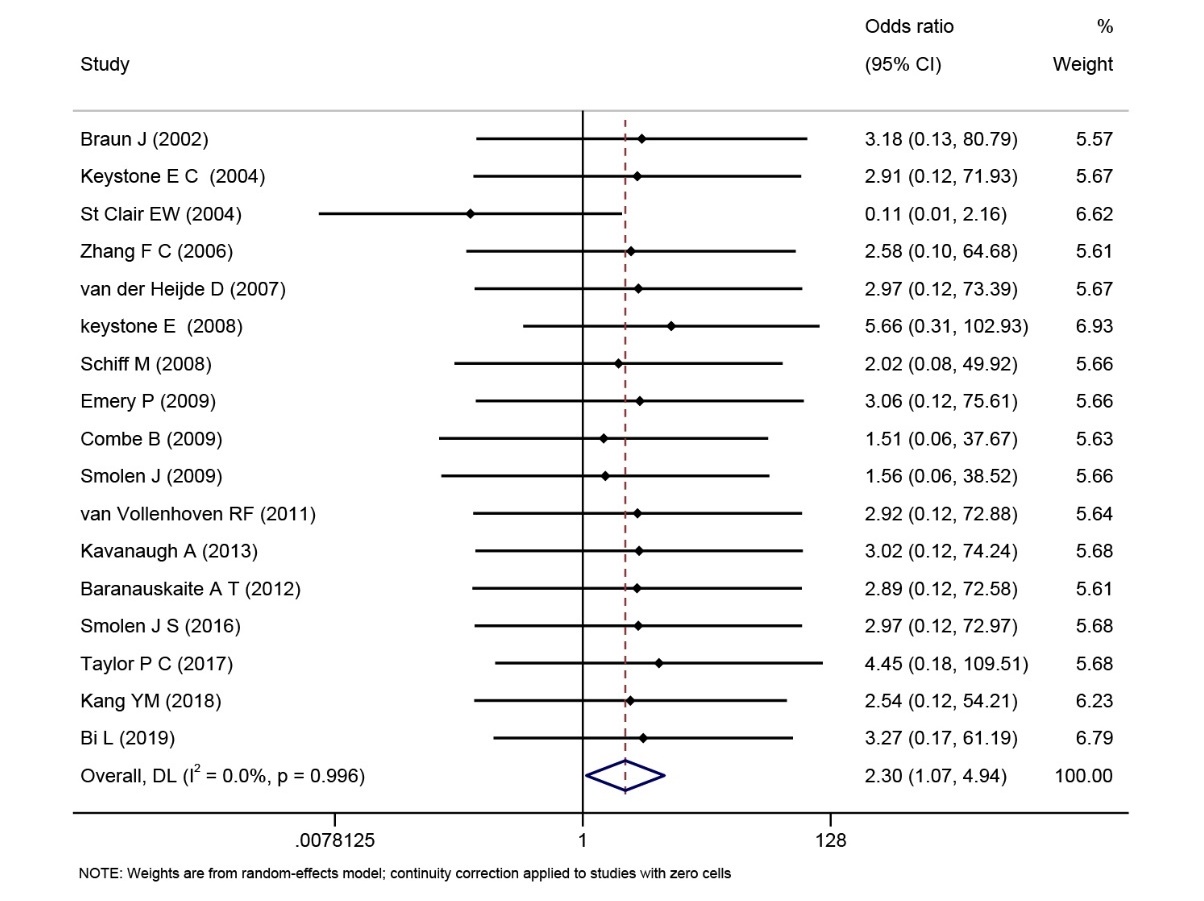
**

**Supplementary Figure S5. The SUCRA probabilities of the tumor necrosis factor-α inhibitors on risk of infections. (A) Serious infection. (B) Any infection.**

**A**


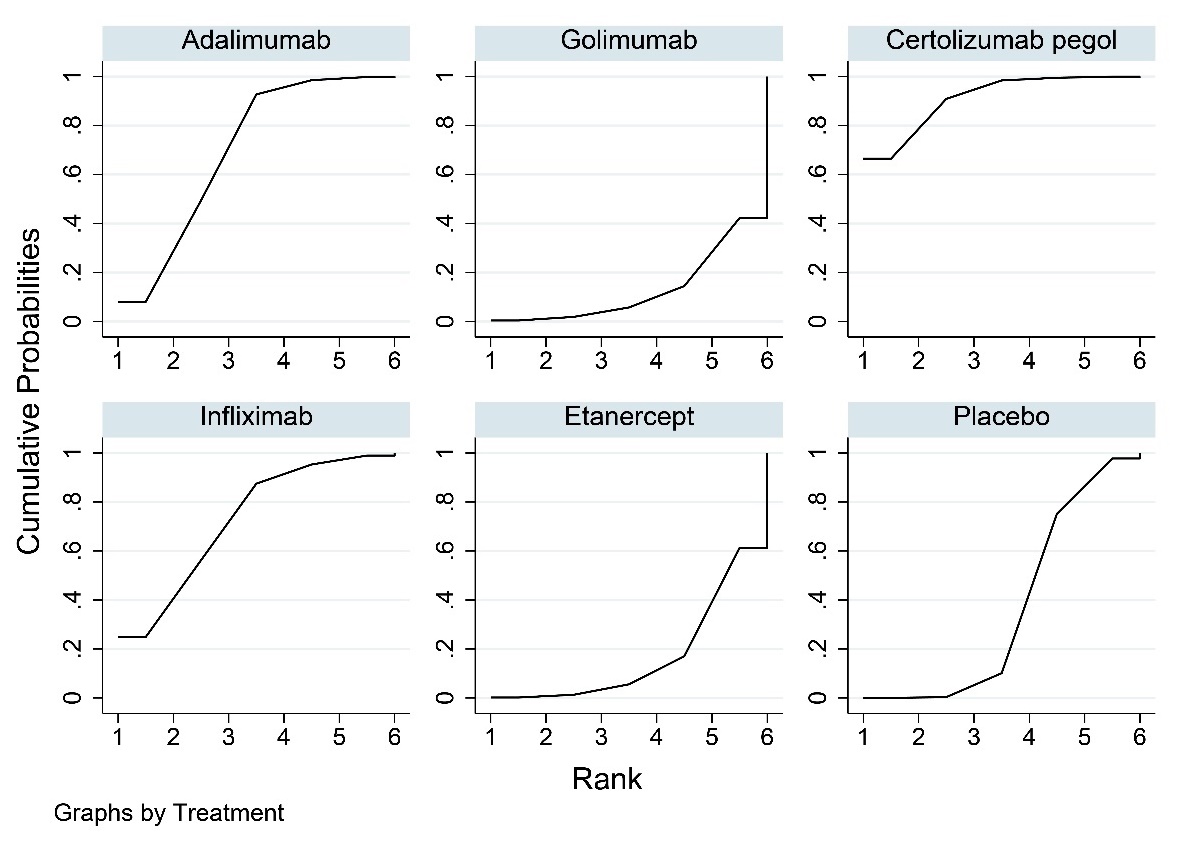


**B**


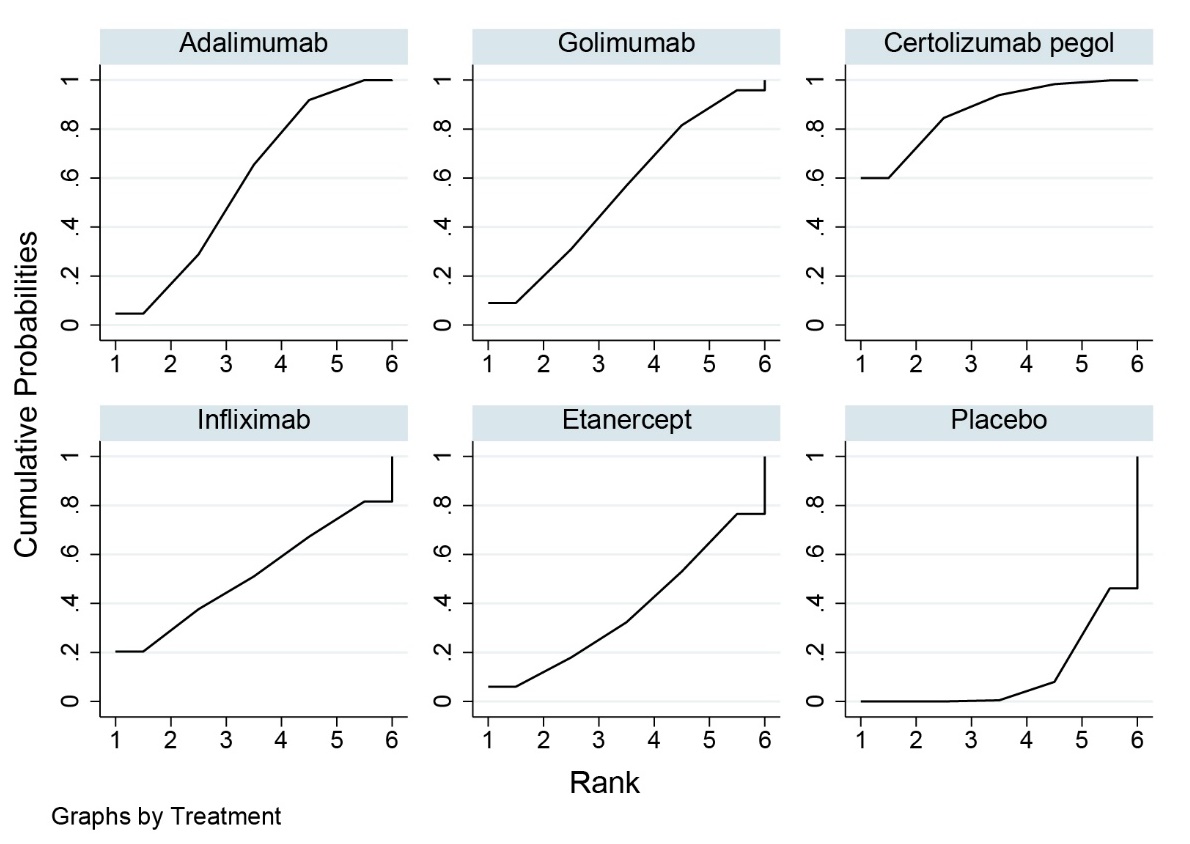


SUCRA represents the likelihood that they are rated as having the highest risk. A higher probability of SUCRA indicates a higher risk of infection.

**Supplementary Table S2.** **Meta-regression of trails’ characteristics on the risk of TNF inhibitors.**

**Table S2.1 Serious infection**

| **Variable** | **Coef.** | **Std. Err.** | **t** | **P>\|t\|** | **95% CI** |
| --- | --- | --- | --- | --- | --- |
| **Age** | 0.060 | 0.399 | 0.15 | 0.882 | (-0.762,0.881) |
| **Background treatment** | -0.632 | 0.660 | -0.96 | 0.347 | (-1.991,0.726) |
| **Follow-up** | -0.388 | 0.340 | -1.14 | 0.265 | (-1.088,0.312) |
| **Inflammatory condition** | 0.542 | 0.820 | 0.66 | 0.514 | (-1.146,2.231) |

**Table S2.2 Any infection**

| **Variable** | **Coef.** | **Std. Err.** | **t** | **P>\|t\|** | **95% CI** |
| --- | --- | --- | --- | --- | --- |
| **Age** | 0.279 | 0.262 | 1.07 | 0.305 | (-0.283,0.841) |
| **Background treatment** | -0.041 | 0.266 | -0.15 | 0.879 | (-0.611,0.529) |
| **Follow-up** | 0.000 | 0.157 | 0.00 | 0.998 | (-0.336,0.337) |
| **Inflammatory condition** | -0.637 | 0.621 | -1.03 | 0.332 | (-1.969,0.695) |

**Table S2.3 Opportunistic infection**

| **Variable** | **Coef.** | **Std. Err.** | **t** | **P>\|t\|** | **95% CI** |
| --- | --- | --- | --- | --- | --- |
| **Age** | -2.161 | 1.714 | -1.26 | 0.334 | (-9.534,5.212) |
| **Follow-up** | -0.167 | 1.788 | -0.09 | 0.934 | (-7.858,7.525) |
| **Inflammatory condition** | 0.583 | 2.257 | 0.26 | 0.820 | (-9.127,10.293) |

**Table S2.4 Tuberculosis**

| **Variable** | **Coef.** | **Std. Err.** | **t** | **P>\|t\|** | **95% CI** |
| --- | --- | --- | --- | --- | --- |
| **Age** | -0.513 | 1.047 | -0.49 | 0.634 | (-2.819,1.792) |
| **Background treatment** | -0.622 | 1.600 | -0.39 | 0.705 | (-4.143,2.899) |
| **Follow-up** | -0.211 | 0.976 | -0.22 | 0.833 | (-2.360,1.938) |
| **Inflammatory condition** | -2.164 | 1.133 | -0.19 | 0.852 | (-2.709,2.275) |

**Table S2.5 Herpes zoster**

| **Variable** | **Coef.** | **Std. Err.** | **t** | **P>\|t\|** | **95% CI** |
| --- | --- | --- | --- | --- | --- |
| **Age** | -0.605 | 1.218 | -0.50 | 0.645 | (-3.987,2.777) |
| **Background treatment** | 0.616 | 1.681 | 0.37 | 0.733 | (-4.052,5.283) |
| **Follow-up** | -0.650 | 1.468 | -0.44 | 0.681 | (-4.725,3.426) |

**Supplementary Table S3.** **Subgroup analysis of trails’ characteristics (P-value) .**

**Table S3.1 Serious infection**

|  | **NO** | **OR(95% CI)** | **P within group** |
| --- | --- | --- | --- |
| **Age** | 51 |  | 0.338 |
| ≤50 years | 21 | 0.92(0.55,1.54) |  |
| ＞50 years | 30 | 1.22(0.94,1.57) |  |
| **Background therapy used** | 30 |  | 0.172 |
| Yes | 26 | 1.29(0.98,1.70) |  |
| No | 4 | 3.01(0.92,9.84) |  |
| **Follow-up** | 52 |  | 0.952 |
| ≤6 months | 36 | 1.14(0.81,1.60) |  |
| ＞6 months | 16 | 1.21(0.83,1.61) |  |
| **Inflammatory condition** | 51 |  | 0.311 |
| RA | 38 | 1.21(0.95,1.54) |  |
| AS | 7 | 0.71(0.21,2.33) |  |
| PsA | 7 | 0.63(0.24,1.64) |  |

**Table S3.2 Any infection**

|  | **NO** | **OR(95% CI)** | **P within group** |
| --- | --- | --- | --- |
| **Age** | 37 |  | 0.728 |
| ≤50 years | 13 | 1.15(0.94,1.42) |  |
| ＞50 years | 24 | 1.20(1.10,1.32) |  |
| **Background therapy used** | 19 |  | 0.659 |
| Yes | 16 | 1.19(1.05,1.36) |  |
| No | 3 | 1.06(0.64,1.75) |  |
| **Follow-up** | 37 |  | 0.782 |
| ≤6 months | 29 | 1.19(1.07,1.31) |  |
| ＞6 months | 8 | 1.22(1.06,1.40) |  |
| **Inflammatory condition** | 35 |  | 0.641 |
| RA | 26 | 1.21(1.11,1.33) |  |
| AS | 8 | 1.25(0.98,1.60) |  |
| PsA | 3 | 1.00(0.65,1.52) |  |

**Table S3.3 Opportunistic infection**

|  | **NO** | **OR (95% CI)** | **P within group** |
| --- | --- | --- | --- |
| **Age** | 6 |  | 0.059 |
| ≤50 years | 2 | 4.46(0.49,40.56) |  |
| ＞50 years | 4 | 0.38(0.10,1.38) |  |
| **Follow-up** | 6 |  | 0.616 |
| ≤6 months | 5 | 0.79(0.24,2.61) |  |
| ＞6 months | 1 | 0.33(0.01,8.13) |  |
| **Inflammatory condition** | 6 |  |  |
| RA | 5 | 0.53(0.16,1.74) | 0.163 |
| PsA | 1 | 6.05(0.25,149.56) |  |

**TableS3.4 Tuberculosis**

|  | **NO** | **OR (95% CI)** | **P within group** |
| --- | --- | --- | --- |
| **Age** | 12 |  | 0.397 |
| ≤50 years | 3 | 2.45 (0.63,9.58) |  |
| ＞50 years | 9 | 1.21(0.49,2.96) |  |
| **Background therapy used** | 8 |  | 0.691 |
| Yes | 6 | 1.68(0.60,4.71) |  |
| No | 2 | 1.01 (0.10,9.79) |  |
| **Follow-up** | 13 |  | 0.413 |
| ≤6 months | 7 | 1.01(0.31,3.32) |  |
| ＞6 months | 6 | 1.90(0.75,4.79) |  |
| **Inflammatory condition** | 13 |  | 0.949 |
| RA | 10 | 1.63(0.73,3.61) |  |
| AS | 1 | 1.58(0.06,41.34) |  |
| PsA | 2 | 0.87(0.02,35.04) |  |

**TableS3.5 Tuberculosis**

|  | **NO** | **OR (95% CI)** | **P within group** |
| --- | --- | --- | --- |
| **Age** | 9 |  | 0.503 |
| ≤50 years | 2 | 2.00(0.44,9.05) |  |
| ＞50 years | 7 | 1.08(0.41,2.88) |  |
| **Background therapy used** | 6 |  | 0.736 |
| Yes | 4 | 1.57(0.49,5.00) |  |
| No | 2 | 1.01(0.10,9.79) |  |
| **Follow-up** | 10 |  | 0.242 |
| ≤6 months | 5 | 0.66(0.16,2.68) |  |
| ＞6 months | 5 | 1.82(0.69,4.79) |  |
| **Inflammatory condition** | 10 |  | 0.307 |
| RA | 8 | 1.56(0.67,3.66) |  |
| AS | 1 | 1.58(0.06,41.34) |  |
| PsA | 1 | 0.14(0.59,2.72) |  |

**Supplementary Table S4. GRADE assessment**

| Outcomes | Comparison | Nature of the evidence | Confidence | Downgrading due to |
| --- | --- | --- | --- | --- |
| Serious infection | Adalimumab vs Placebo | Mixed | ⨁⨁◯◯  Low | Indirectness, Imprecision |
|  | Golimumab vs Placebo | Direct | ⨁⨁⨁◯  Moderate | Imprecision |
|  | Certolizumab pegol vs Placebo | Mixed | ⨁⨁⨁◯  Moderate | Indirectness |
|  | Infliximab vs Placebo | Direct | ⨁⨁◯◯  Low | Imprecision, Inconsistency |
|  | Etanercept vs Placebo | Direct | ⨁⨁⨁◯  Moderate | Imprecision |
|  | Adalimumab vs Golimumab | Indirect | ⨁⨁⨁◯  Moderate | Imprecision |
|  | Adalimumab vs Certolizumab | Mixed | ⨁⨁⨁◯  Moderate | Indirectness |
|  | Adalimumab vs Infliximab | Indirect | ⨁⨁⨁◯  Moderate | Imprecision |
|  | Adalimumab vs Etanercept | Indirect | ⨁⨁⨁◯  Moderate | Imprecision |
|  | Golimumab vs Certolizumab | Indirect | ⨁⨁⨁◯  Moderate | Imprecision |
|  | Golimumab vs Infliximab | Indirect | ⨁⨁⨁◯  Moderate | Imprecision |
|  | Golimumab vs Etanercept | Indirect | ⨁⨁⨁◯  Moderate | Imprecision |
|  | Certolizumab vs Infliximab | Indirect | ⨁⨁⨁◯  Moderate | Imprecision |
|  | Certolizumab vs Etanercept | Indirect | ⨁⨁⨁◯  Moderate | Imprecision |
|  | Infliximab vs Etanercept | Indirect | ⨁⨁⨁◯  Moderate | Imprecision |
|  | Ranking of treatments |  | ⨁⨁⨁◯  Moderate | Imprecision |
| Any infection | Adalimumab vs Placebo | Direct | ⨁⨁⨁◯  Moderate | Imprecision |
|  | Golimumab vs Placebo | Direct | ⨁⨁⨁◯  Moderate | Imprecision |
|  | Certolizumab pegol vs Placebo | Direct | ⨁⨁⨁◯  Moderate | Imprecision |
|  | Infliximab vs Placebo | Direct | ⨁⨁◯◯  Low | Inconsistency, Imprecision |
|  | Etanercept vs Placebo | Direct | ⨁⨁◯◯  Low | Inconsistency, Imprecision |
|  | Adalimumab vs Golimumab | Indirect | ⨁⨁⨁◯  Moderate | Imprecision |
|  | Adalimumab vs Certolizumab | Indirect | ⨁⨁⨁◯  Moderate | Imprecision |
|  | Adalimumab vs Infliximab | Indirect | ⨁⨁⨁◯  Moderate | Imprecision |
|  | Adalimumab vs Etanercept | Indirect | ⨁⨁⨁◯  Moderate | Imprecision |
|  | Golimumab vs Certolizumab | Indirect | ⨁⨁⨁◯  Moderate | Imprecision |
|  | Golimumab vs Infliximab | Indirect | ⨁⨁⨁◯  Moderate | Imprecision |
|  | Golimumab vs Etanercept | Indirect | ⨁⨁⨁◯  Moderate | Imprecision |
|  | Certolizumab vs Infliximab | Indirect | ⨁⨁⨁◯  Moderate | Imprecision |
|  | Certolizumab vs Etanercept | Indirect | ⨁⨁⨁◯  Moderate | Imprecision |
|  | Infliximab vs Etanercept | Indirect | ⨁⨁⨁◯  Moderate | Imprecision |
|  | Ranking of treatments |  | ⨁⨁⨁◯  Moderate | Imprecision |
| Opportunistic infection | Etanercept vs Placebo | Direct | ⨁⨁⨁◯  Moderate | Imprecision |
|  | Infliximab vs Placebo | Direct | ⨁⨁◯◯  Low | Inconsistency, Imprecision |
|  | Adalimumab vs Placebo | Direct | ⨁⨁⨁◯  Moderate | Imprecision |
| Herpes zoster | Infliximab vs Placebo | Direct | ⨁⨁⨁◯  Moderate | Imprecision |
|  | Adalimumab vs Placebo | Mixed | ⨁⨁◯◯  Low | Indirectness, Imprecision |
|  | Etanercept vs Placebo | Direct | ⨁⨁⨁◯  Moderate | Imprecision |
|  | Golimumab vs Placebo | Direct | ⨁⨁⨁◯  Moderate | Imprecision |
|  | Certolizumab pegol vs Placebo | Mixed | ⨁⨁⨁◯  Moderate | Indirectness |
|  | Adalimumab vs Certolizumab | Mixed | ⨁⨁⨁◯  Moderate | Indirectness |
| Tuberculosis | Infliximab vs Placebo | Direct | ⨁⨁⨁◯  Moderate | Imprecision |
|  | Adalimumab vs Placebo | Mixed | ⨁⨁◯◯  Low | Indirectness, Imprecision |
|  | Etanercept vs Placebo | Direct | ⨁⨁⨁◯  Moderate | Imprecision |
|  | Certolizumab pegol vs Placebo | Mixed | ⨁⨁◯◯  Low | Indirectness, Imprecision |
|  | Adalimumab vs Certolizumab | Mixed | ⨁⨁◯◯  Low | Indirectness, Imprecision |

^1^Risk of bias: We performed sensitivity analyses that did not identify any studies with a high risk of bias, indicating that there is no need to downgrade because of risk of bias.

^2^Indirectness: Indirect comparisons always warrant rating down by one level in quality of evidence.

^3^Inconsistency: By comparing the effect estimates of the direct and indirect results after network splitting, we observed consistency across all comparisons.

^4^Imprecision: For odds ratio that cross the null-effect threshold (OR=1) we downgraded with one. If the threshold is not crossed we did not downgrade.

**Supplementary Table S5. Checklist of the PRISMA extension for network meta-analysis**

| **Section/Topic** | **Item #** | **Checklist Item** | **Reported on Page #** |
| --- | --- | --- | --- |
| **TITLE** |  |  |  |
| Title | 1 | Identify the report as a systematic review *incorporating a network meta-analysis (or related form of meta-analysis).* |  |
|  |  |  |  |
| **ABSTRACT** |  |  |  |
| Structured summary | 2 | Provide a structured summary including, as applicable:  **Background:** main objectives  **Methods:** data sources; study eligibility criteria, participants, and interventions; study appraisal; and *synthesis methods, such as network meta-analysis.*  **Results:** number of studies and participants identified; summary estimates with corresponding confidence/credible intervals; *treatment rankings may also be discussed. Authors may choose to summarize pairwise comparisons against a chosen treatment included in their analyses for brevity.*  **Discussion/Conclusions:** limitations; conclusions and implications of findings.  **Other:** primary source of funding; systematic review registration number with registry name. |  |
|  |  |  |  |
| **INTRODUCTION** |  |  |  |
| Rationale | 3 | Describe the rationale for the review in the context of what is already known*, including mention of why a network meta-analysis has been conducted.* |  |
| Objectives | 4 | Provide an explicit statement of questions being addressed, with reference to participants, interventions, comparisons, outcomes, and study design (PICOS). |  |
|  |  |  |  |
| **METHODS** |  |  |  |
| Protocol and registration | 5 | Indicate whether a review protocol exists and if and where it can be accessed (e.g., Web address); and, if available, provide registration information, including registration number. |  |
| Eligibility criteria | 6 | Specify study characteristics (e.g., PICOS, length of follow-up) and report characteristics (e.g., years considered, language, publication status) used as criteria for eligibility, giving rationale. *Clearly describe eligible treatments included in the treatment network, and note whether any have been clustered or merged into the same node (with justification).* |  |
| Information sources | 7 | Describe all information sources (e.g., databases with dates of coverage, contact with study authors to identify additional studies) in the search and date last searched. |  |
| Search | 8 | Present full electronic search strategy for at least one database, including any limits used, such that it could be repeated. | Supplementary Table S1 |
| Study selection | 9 | State the process for selecting studies (i.e., screening, eligibility, included in systematic review, and, if applicable, included in the meta-analysis). |  |
| Data collection process | 10 | Describe method of data extraction from reports (e.g., piloted forms, independently, in duplicate) and any processes for obtaining and confirming data from investigators. |  |
| Data items | 11 | List and define all variables for which data were sought (e.g., PICOS, funding sources) and any assumptions and simplifications made. |  |
| **Geometry of the network** | **S1** | Describe methods used to explore the geometry of the treatment network under study and potential biases related to it. This should include how the evidence base has been graphically summarized for presentation, and what characteristics were compiled and used to describe the evidence base to readers. |  |
| Risk of bias within individual studies | 12 | Describe methods used for assessing risk of bias of individual studies (including specification of whether this was done at the study or outcome level), and how this information is to be used in any data synthesis. |  |
| Summary measures | 13 | State the principal summary measures (e.g., risk ratio, difference in means). *Also describe the use of additional summary measures assessed, such as treatment rankings and surface under the cumulative ranking curve (SUCRA) values, as well as modified approaches used to present summary findings from meta-analyses.* |  |
| Planned methods of analysis | 14 | Describe the methods of handling data and combining results of studies for each network meta-analysis. This should include, but not be limited to:   - *Handling of multi-arm trials;* - *Selection of variance structure;* - *Selection of prior distributions in Bayesian analyses; and* - *Assessment of model fit.* |  |
| **Assessment of Inconsistency** | **S2** | Describe the statistical methods used to evaluate the agreement of direct and indirect evidence in the treatment network(s) studied. Describe efforts taken to address its presence when found. |  |
| Risk of bias across studies | 15 | Specify any assessment of risk of bias that may affect the cumulative evidence (e.g., publication bias, selective reporting within studies). |  |
| Additional analyses | 16 | Describe methods of additional analyses if done, indicating which were pre-specified. This may include, but not be limited to, the following:   - Sensitivity or subgroup analyses; - Meta-regression analyses; - *Alternative formulations of the treatment network; and* - *Use of alternative prior distributions for Bayesian analyses (if applicable).* |  |
|  |  |  |  |
| **RESULTS†** |  |  |  |
| Study selection | 17 | Give numbers of studies screened, assessed for eligibility, and included in the review, with reasons for exclusions at each stage, ideally with a flow diagram. | Figure 1 |
| **Presentation of network structure** | **S3** | Provide a network graph of the included studies to enable visualization of the geometry of the treatment network. | Figure 2 |
| **Summary of network geometry** | **S4** | Provide a brief overview of characteristics of the treatment network. This may include commentary on the abundance of trials and randomized patients for the different interventions and pairwise comparisons in the network, gaps of evidence in the treatment network, and potential biases reflected by the network structure. |  |
| Study characteristics | 18 | For each study, present characteristics for which data were extracted (e.g., study size, PICOS, follow-up period) and provide the citations. |  |
| Risk of bias within studies | 19 | Present data on risk of bias of each study and, if available, any outcome level assessment. | Supplementary Figure S1 |
| Results of individual studies | 20 | For all outcomes considered (benefits or harms), present, for each study: 1) simple summary data for each intervention group, and 2) effect estimates and confidence intervals. *Modified approaches may be needed to deal with information from larger networks.* | Supplementary Figure S2 |
| Synthesis of results | 21 | Present results of each meta-analysis done, including confidence/credible intervals. *In larger networks, authors may focus on comparisons versus a particular comparator (e.g. placebo or standard care), with full findings presented in an appendix. League tables and forest plots may be considered to summarize pairwise comparisons.* If additional summary measures were explored (such as treatment rankings), these should also be presented. | Figure 3, Supplementary Figure S5 |
| **Exploration for inconsistency** | **S5** | Describe results from investigations of inconsistency. This may include such information as measures of model fit to compare consistency and inconsistency models, *P* values from statistical tests, or summary of inconsistency estimates from different parts of the treatment network. |  |
| Risk of bias across studies | 22 | Present results of any assessment of risk of bias across studies for the evidence base being studied. | Supplementary Figure S1 |
| Results of additional analyses | 23 | Give results of additional analyses, if done (e.g., sensitivity or subgroup analyses, meta-regression analyses*, alternative network geometries studied, alternative choice of prior distributions for Bayesian analyses,* and so forth). | Supplementary Table S2, Supplementary Table S3 |
|  |  |  |  |
| **DISCUSSION** |  |  |  |
| Summary of evidence | 24 | Summarize the main findings, including the strength of evidence for each main outcome; consider their relevance to key groups (e.g., healthcare providers, users, and policy-makers). |  |
| Limitations | 25 | Discuss limitations at study and outcome level (e.g., risk of bias), and at review level (e.g., incomplete retrieval of identified research, reporting bias). *Comment on the validity of the assumptions, such as transitivity and consistency. Comment on any concerns regarding network geometry (e.g., avoidance of certain comparisons).* |  |
| Conclusions | 26 | Provide a general interpretation of the results in the context of other evidence, and implications for future research. |  |
|  |  |  |  |
| **FUNDING** |  |  |  |
| Funding | 27 | Describe sources of funding for the systematic review and other support (e.g., supply of data); role of funders for the systematic review. This should also include information regarding whether funding has been received from manufacturers of treatments in the network and/or whether some of the authors are content experts with professional conflicts of interest that could affect use of treatments in the network. |  |

PICOS = population, intervention, comparators, outcomes, study design.

* Text in italics indicateS wording specific to reporting of network meta-analyses that has been added to guidance from the PRISMA statement.

† Authors may wish to plan for use of appendices to present all relevant information in full detail for items in this section.
